# AI-Driven Mental Health Support for Caregivers of Individuals With Alzheimer Disease: Systematic Literature Review and Development of a Conceptual Framework

**DOI:** 10.2196/79973

**Published:** 2026-03-06

**Authors:** Syeda Umme Salma, Chandra Rekha Renduchintala, Isa Siddique, Evelina Sterling, Sweta Sneha, Nazmus Sakib

**Affiliations:** 1 Kennesaw State University Marietta, GA United States

**Keywords:** Alzheimer disease, caregivers, mental health, artificial intelligence, personalized care, explainable artificial intelligence, mHealth, machine learning, real time monitoring

## Abstract

**Background:**

Caregivers supporting individuals with Alzheimer disease and related dementias (AD/ADRD) frequently encounter prolonged emotional strain, psychological distress, and social isolation, yet their needs are largely overlooked in current technological and clinical interventions. The special routines and obligations of caregivers of individuals with AD/ADRD are frequently not well-suited to the many artificial intelligence–driven (AI-driven) mental health solutions that are currently available. This reveals a critical need for sophisticated, customized solutions created especially to help the mental health of caregivers for patients with AD/ADRD.

**Objective:**

To address the existing limitations of personalized mental health interventions, we aimed to identify existing literature on personalized mental health interventions using AI for specific purposes and to develop a new framework for the caregivers of individuals with AD/ADRD.

**Methods:**

We followed an iterative approach to design the new framework. First, we did a systematic literature review of current literature to identify data analysis, AI methods, and personalized interventions. Second, we focused on the underlying gaps of this research, and by synthesizing our findings from the review, we proposed a conceptual framework.

**Results:**

The systematic literature review identified 73 unique results, and from external sources, we found 3 unique potential papers. Of these, 28 papers were eligible for inclusion, on which we performed our analysis. Based on the findings, we developed a new conceptual framework with 3 special features that are specifically for caregivers of patients with AD/ADRD. The 3 unique features are a personalized daily routine scheduler, which will take both patients with AD/ADRD and caregiver’s information to make it personalized, a daily reward system to keep patients motivated, and an educational repository to get the bite-sized knowledge for the lesson of handling patients in an efficient manner and taking care of one’s own mental health.

**Conclusions:**

The proposed framework provides a chance for caregivers to receive mental health care, which will be personalized. The framework is developed with more updated methods than existing approaches, with a lack of personalization in this sector. This framework can be implemented with a goal of personalization and explainable approaches and can undergo further iterations to ensure it is appropriate for specific purposes.

## Introduction

Alzheimer disease and related dementias (AD/ADRD) demand care to give the patients full-time support, and in that case, formal or informal caregivers play a very important role. However, the demand for caregivers of individuals with AD/ADRD is currently surging with the older population who were born between 1946 and 1964 (also known as the baby boomer generation [1]). For instance, according to the Alzheimer Association [2], in 2024, there were approximately 7 million Americans aged 65 years and older experiencing AD, and the number of patients might double by 2060. Therefore, this baby boomer generation is placing a high demand on caregivers as they are entering the high-risk age bracket for AD. This sudden need creates a critical shortage of formal caregivers and emotional challenges among informal caregivers, such as family members or loved ones.

Though the demand for caregivers is increasing rapidly, their number has not increased accordingly, burdening them with workloads beyond their capacity. This scarcity might put pressure on caregivers, especially informal caregivers who often bear the burden quietly in this situation. For instance, in the United States alone, more than 11 million unpaid caregivers offer 18 billion hours of care, worth nearly US $340 billion yearly, to patients with AD/ADRD, according to the Alzheimer Association [2]. The study by Sallim et al [3] found that roughly 34% of caregivers experience depression, while 43.6% report anxiety. Apart from that, caregivers mostly have to deal with sedentary lifestyles, sleep deprivation, social isolation, and foretaste grief, which are not even treated by professionals due to time and financial constraints [4]. Therefore, there is a pressing need for a change in attention towards the mental health support system customized for caregivers of individuals with AD/ADRD, which will allow them to manage their burden and have some time for themselves to work on their well-being.

Caregivers of individuals with AD/ADRD need a system that will be personalized depending on the stage of patients with AD/ADRD, the caregiver’s own preferences, and background. There are various digitally accessible mental health supports for caregivers of individuals with AD/ADRD, such as informational portals and educational resources [5,6], therapy and counseling services [7], peer-led support groups [8,9], and some digital tools and mobile apps [10-12]. The purpose of these interventions is to improve the mental health of caregivers for patients with AD/ADRD. However, existing interventions have some limitations, too. For instance, some mobile apps offer limited interactivity, minimal emotional support integration, and static content delivery, which lacks adaptive learning or personalization based on caregiver experience level or stress indicators [10,12]. Besides, some interventions [11], require smart home infrastructure, which may not be accessible to low-income or older caregivers.

In order to provide more background of our study, we group the technologies discussed in this paper into six components: (1) natural language processing (NLP): analyzes text (eg, notes, messages, and forum posts) to detect topics, symptoms, or risk; (2) recommender systems and personalization policies: select the “next best” tip, task, or content and adjust timing based on user state and preferences; (3) conversational agents: rule-based or large language model chatbots that deliver check-ins or guidance; (4) multimodal sensing: passive phone/wearable signals (eg, sleep, steps, and heart rate) optionally combined with brief ecological momentary assessments (EMA); (5) modeling approaches: classical machine learning such as logistic regression, random forests and deep learning, for example, recurrent/transformer networks used for prediction; and (6) explainability and deployment practices: methods that show why a model made a suggestion such as feature importance, saliency and operational safeguards (calibration, drift monitoring, and privacy). We use this taxonomy throughout the paper, such as research question (RQ) 1 (RQ1) addresses data and processing, RQ2 addresses personalization and monitoring, and RQ3 addresses explainability and clinical integration.

To make a system more acceptable to users, certain artificial intelligence (AI) design guidelines should be followed [13]. Amershi et al [13] presented 18 guidelines to ensure AI-infused apps are understandable, trustworthy, fair, and usable. Among the guidelines, guideline G11 is about making clear why the system did what it did. These explanations are highly important for users to trust the system and have transparency between users and the system. Though this guideline is crucial in creating human-centric AI systems, most of the recent caregiver apps [10-12] do not follow the guideline of explainable AI. Moreover, according to Jung et al [14], the aspect of explainability is vital for fostering trust and ensuring that both caregivers and clinicians can comprehend and effectively apply AI-generated recommendations.

**Figure 1:**
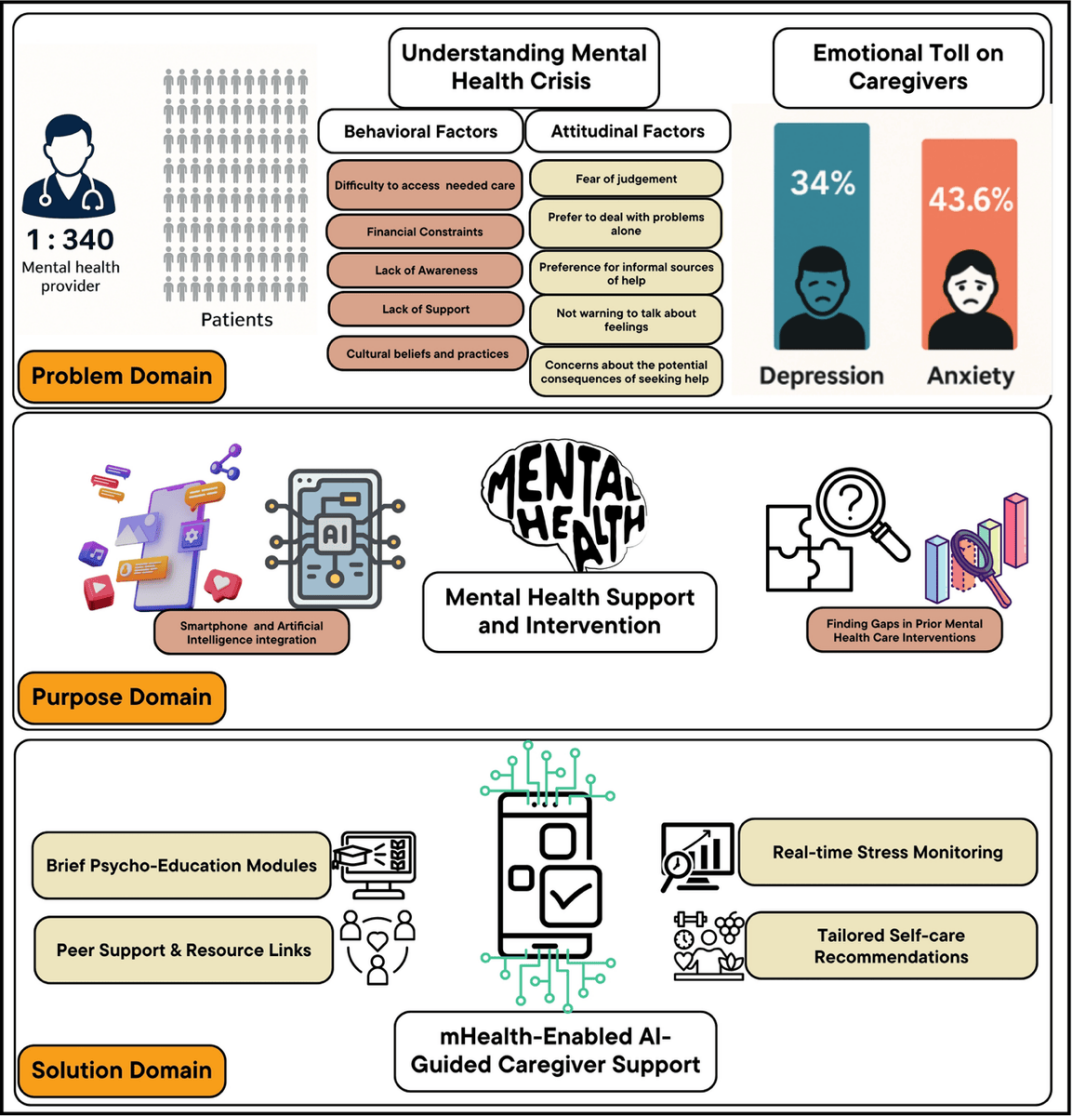
Problem identification to solution. AI: artificial intelligence; mHealth: mobile health.

**Figure 2:**
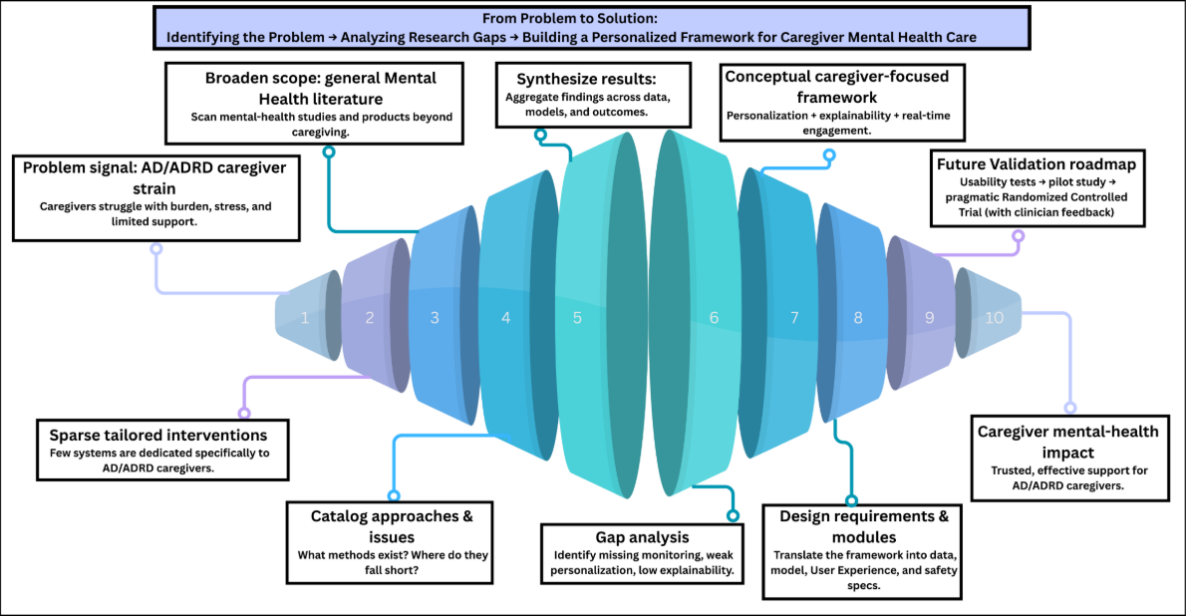
Evidence to impact: designing a solution for AD/ADRD caregivers. AD/ADRD: Alzheimer disease and related dementias.

Rather than proceeding directly to the design of a caregiver-centered intervention, our study begins with a systematic review of personalized mental health technologies to establish a comprehensive understanding of current trends, limitations, and opportunities. This approach allows us to learn from and build upon the broader mental health innovation landscape before narrowing our focus to caregiving for individuals with AD/ADRD. The overview of this study is shown in Figures 1 and 2. To guide this review, we developed the following RQs:

- **RQ1**: What types of data are used for detecting early warning signs of mental disorders, and how are these data collected and processed?
- **RQ2**: What types of AI models are used in current depression monitoring systems, and what limitations hinder their effectiveness in real-world practice?
- **RQ3**: What usability and design challenges arise in implementing explainable human-AI interaction systems for mental health care?

By systematically reviewing these areas, we aim to extract key insights that will guide the design of our caregiver-focused mental health intervention framework. In this study, we have discussed the findings of the review in the Results section. Briefly, we found heterogeneous types of data sources, and among them, clinical trials and mobile apps were the most adapted. Besides, real-time monitoring was highly rare and even poorly reported. Personalization and explainability were present in some of the studies. However, those were inconsistently defined and rarely transparent. To summarize the findings, the identified gaps indicate the need for hybrid sensing, such as passive data augmented with brief self-reports, policy-transparent personalization, and clinician-oriented explanations to enable trustworthy adoption. Based on our findings, we are proposing a conceptual framework for personalized AI-driven mental health intervention. This framework is designed to support dynamic treatment personalization, explainability, and real-time user engagement, particularly for caregivers of Alzheimer-affected individuals. We derived the caregiver-specific components from proven dementia-caregiver interventions (psychoeducation or skills training, stress reduction, counseling, or peer support) and standard outcome frameworks (burden, mood, coping, and role functioning) [15-19]. We then translated these needs into low-burden digital features such as hybrid sensing with brief check-ins, just-in-time support, guided by Human-AI Interaction and Just-in-Time Adaptive Interventions principles [13,20,21]. The framework is broadly discussed in the discussion section.

## Methods

### Database Selection and Search Strategy

In this systematic review, we followed the PRISMA (Preferred Reporting Items for Systematic Reviews and Meta-Analyses) guidelines (Multimedia Appendix 1). The review was started in February 2025 using the 5 main databases, such as PubMed, Google Scholar, IEEE Xplore, ScienceDirect, and Scopus, following specific search queries (provided in Multimedia Appendix 2). Only peer-reviewed publications published between January 2020 and February 2025 were included in the search parameters. To maintain methodological integrity, we have only included original research articles that were written in English language. In this review, book chapters, structured or unstructured reviews, observational notes, and position papers were not included.

The search string (Multimedia Appendix 2) was carefully designed to fit the emphasis of our work. Interventions of our interest included the use of smart devices and AI-enabled or precision medicine approaches for mental health. Moreover, we considered research indicating promise for personalized treatment. The study excluded studies that are not connected to mental health treatment or AI-assisted solutions.

### Study Selection

One team member (SU) performed the initial screening of studies by title. Then, 2 individual reviewers (SU) and (CR) were given the task of carrying out the screening, determining eligibility, and managing the inclusion procedure. After careful consideration, any discrepancies between the 2 teams were fixed through agreement. Both reviewers independently then screened the potential studies by title and abstract. A third team (IS) was consulted to make the final decision in cases where an agreement could not be reached. From February to March 20, 2025, the screening procedure was carried out following the inclusion and exclusion criteria. Studies that did not relate to AI-driven personalized interventions for mental health treatment were not considered. Additionally, articles that focused on other medical conditions, were not subject to peer review, or were editorials, interviews, or position papers, were not included.

### Selection and Inclusion of Studies

The PRISMA flow diagram summarizes the study selection process. The initial search yielded a total of 76 citations, from which 13 duplicates were removed. The remaining records were screened based on their titles and abstracts, leading to the exclusion of 15 citations that did not meet the inclusion criteria. The remaining 48 studies were then subjected to full-text review, during which 3 papers were excluded. The primary reasons for exclusion included the use of ineligible manuscript types such as published abstracts, studies that focused on mental health treatment without addressing personalized care or precision medicine, and papers that did not involve the application of machine learning or AI. After the screening process, a total of 28 studies were included in the final review.

### Data Extraction

For eligible studies, data were extracted from the studies by 2 teams (IS and CR), and the whole process was reviewed by a third team (SU). Data were extracted from the potential studies by following the RQs. First, information about data sources, data collection methods, data types, preprocessing techniques, and AI models used in the studies was extracted from the reviewed studies. Second, both teams (IS and CR) gathered information about AI methodologies and approaches, real-time monitoring scopes, personalization strategies, intervention models, critical influencing factors, challenges in AI implementations, target population and user groups, and associated mental health conditions. Third, insights were gathered on AI explanation methods (including explainability and interpretability techniques), system design and implementation challenges, trade-offs between model performance and interpretability, clinician-centered barriers and real-world integrations, as well as the impact of AI on clinical decision-making and patient outcomes. After every step, the analysis was evaluated and fine-tuned by another team (SU).

### Developing a Conceptual Framework

The team members of this research analyzed the findings of the review. During our analysis, we emphasized more on real time monitoring tools, personalized approaches, and AI models used. This thorough analysis helped us to develop our comprehensive conceptual framework.

## Results

### Overview

**Figure 3:**
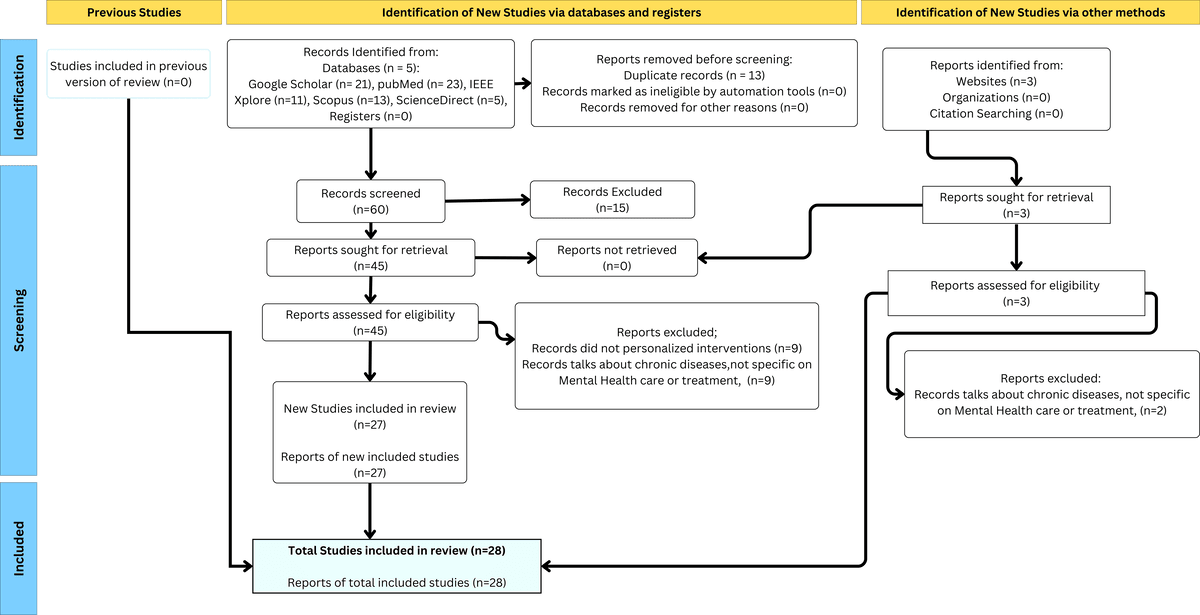
PRISMA (Preferred Reporting Items for Systematic Reviews and Meta-Analyses) flowchart illustrating the inclusion and exclusion of studies.

We analyzed the included studies (Figure 3) and organized the core findings based on RQs. The findings for our RQ1, RQ2, and RQ3 can be found in Multimedia Appendices 3-5 [22-49], consequently. In addition, the shortcomings of single approaches and what complementary methods can be adopted in this case are shown in Table 1.

**Table 1:** Single-approach limitations and recommended complementary methods.

Single approach	What it captures well	What it misses	Common failure modes	Complementary inputs/interventions	Why it fills the gap	Implementation note
Self-report only (mood check-ins, brief surveys) [33,34]	Subjective state, intent, symptom nuance	Day-to-day fluctuations, noncompliance periods, social desirability bias	Sparse/late entries, recall bias [50,51]	Passive sensing (sleep/steps/HR^a^), app usage logs [52]	Objective, continuous context; flags when self-report is absent	Use opt-in sensing; prompt when passives drift from reported mood
Passive wearable only (HRV^b^, sleep, steps) [24,25,35,37,41,42]	Physiology, routines, nocturnal patterns	Cognition, emotion triggers, caregiver burden context	Sensor dropouts, artifacts, poor specificity [53,54]	Just-in-time self-report, short EMA^c^, text notes [20]	Labels events/feelings that physiology cannot disambiguate	Micro-prompts (≤10 s) after anomalous windows
Smartphone/app logs only (usage, taps, screen time) [40,41,43]	Engagement, adherence, micro-routines	Clinical severity, affect, reasons for lapses	“Silent churn”; high noise from noncare tasks [55,56]	Brief affect scale, care task checklist, physiology [57]	Links behavior to state; separates care versus noncare use	Tag care-related screens; couple with weekly PHQ-4^d^
Social media/text only (Reddit, notes) [30,49]	Linguistic markers of stress, topics	Physical burden, sleep, adherence	Topic drift, platform bias, performative posts [58]	Wearables + care task telemetry [59]	Validates language with lived routines	Limit domain shift; time-align posts to passives
Chatbot-only (dialogue transcripts) [28,30]	Perceived support, coping language	Real adherence, off-chat distress	Hawthorne effect; “green-dot” compliance [60]	Background passives, periodic scales [57]	Detects off-session deterioration	Log safety phrases; escalate if passives worsen
Rule-based personalization (fixed heuristics) [22,24,26,27,40,41,48]	Interpretability, safety constraints	Individual adaptation, nonstationarity	Stagnation; alert fatigue [61,62]	Bandits/RL^e^-lite + preference signals [63]	Learns timing/content per user	Start conservative; log policy updates
Classical machine learning on small samples [22-24,35,36,40,44-47]	Stability on tabular data	Complex temporal dynamics	Underfit, brittle thresholds [64]	Sequence models + data augmentation [65]	Captures trajectories; reduces variance	Time-aware CV^f^; report calibration
Deep learning only (black-box) [30,31,39,43-45]	Nonlinear patterns, multimodal fusion	Clinician trust, auditability	Opaque errors; spurious cues [66]	Post-hoc XAI^g^ + policy logging + calibration [67]	Explanations, traceability, reliable probabilities	Tiered transparency for high-stakes prompts
EHR^h^/clinic data only (retrospective) [23]	Diagnoses, meds, visits	Home context, daily stressors	Label noise; delayed signals [68,69]	Home passives + care tasks + short EMAs [70,71]	Fills between visits; real-time risk signals	Federate; de-identify; align timelines
Scheduled questionnaires only (weekly/biweekly) [22,33]	Comparable scales over time	In-between episodes, burden spikes	Missed surveys; ceiling effects [50]	Event-triggered micro-prompts + passives [20]	JIT^i^ capture during anomalies	Use anomaly detectors to gate EMAs

^a^HR: heart rate.

^b^HRV: heart rate variability.

^c^EMA: ecological momentary assessment.

^d^PHQ-4: 4-item Patient Health Questionnaire.

^e^RL: reinforcement learning.

^f^CV: cross-validation.

^g^XAI: explainable AI.

^h^EHR: electronic health record.

^i^JIT: just-in-time.

### RQ1: Data Sources, Collection Methods, and Data Processing Techniques

#### Study Designs and Data Sources

The included studies reflect a diverse range of data sources, with the majority drawn from clinical trials, which accounted for 9 out of 28 (32.1%) studies. Mobile apps and smartphone-based systems represented 5 out of 28 (17.9%) studies, including platforms such as Meru Health, Moodie, MUBS, and Foundations, which captured self-reported mood tracking and passive sensor data. Open-source and social media datasets, including data from platforms like Reddit, Kaggle, and GitHub, appeared in 2 out of 28 (7.1%) studies. Wearable devices, such as smartwatches integrated with apps (eg, BrainE), were used in 1 (3.6%) study, as were AI-based mental health chatbots (eg, SAAC), structured surveys and interviews, and outpatient clinical data—each contributing 1 (3.6%) study. The remaining 8 out of 28 (28.6%) studies were classified as other or unspecified, comprising multimodal datasets, broadly described data sources, or those with insufficient detail to assign a specific category.

#### Collection Methods and Feature Extraction

The reviewed studies used a variety of data collection methods, including clinical trials in 3 (10.7%) studies [22,27,29], mobile apps and smartphone-based systems in 2 (7.1%) studies [40,41], open-source or social media data in 2 (7.1%) studies [30,49], outpatient clinical data in 1 (3.6%) study [23], surveys and interviews in 6 (21.4%) studies [31,34,35,38,39,48], feature extraction commonly included demographics and psychosocial traits in 9 (32.1%) studies [22,33,34,38-41,46,48], sentiment and NLP-based features in 3 (10.7%) studies [31,32,42], neurological and physiological metrics in 4 (14.2%) studies [24,25,35,37], while only 2 (7.1%) studies [23,29] focused on model accuracy and prediction.

#### Preprocessing and Integration Techniques

The reviewed studies used a wide range of preprocessing and integration methods. About 42.9% (12 out of 28) of the studies [22,24,25,27,32-35,37,39-41] used at least one explicit preprocessing step. These steps included cleaning the data and dealing with missing values [33,34,37], normalization, standardization, or encoding techniques [25,32,35,39,40], and feature engineering, which included dichotomizing variables or extracting logs [27,41]. There were also mentions of advanced pipelines to keep data from leaking during cross-validation [24,25]. About 28.6% (8 out of 28) of studies [22,25,27,33,34,37,40,41] talked about combining multiple datasets or making different metrics work together. This included combining data from different demographics [22,33], wearable and app-based logs [40,41], and multimodal clinical sources [25,27,34,37]. The other studies (20/28, 71.4%) [23,24,26,28-32,35,36,38,39,42-49] either used datasets from only one source or did not explain how they combined the data. These results show that there are big differences in how the literature reports on preprocessing transparency and integration strategies.

### RQ2: Personalization Strategies and Real-Time AI-Driven Monitoring

#### Real-Time Monitoring Scope

The analysis of real-time monitoring in AI-driven mental health interventions was one of the primary goals of this review. In this review, 3 (10.7%) studies [35,41,42] used real-time digital monitoring, such as through wearables or mobile apps; 2 (7.1%) studies [33,34] used scheduled mood or symptom questionnaires. Two (7.1%) studies used self-reported or passive sensor monitoring [40,43]. Two (7.1%) studies [37,45] used hybrid or unconventional methods, and 1 (3.6%) study reported periodic clinical follow-ups [25]. However, one significant gap in continuous tracking was highlighted by the fact that 18 out of 28 (64.3%) studies [22-24,26-32,36,38,39,44,46-49] either did not have any monitoring component or did not clearly report it. These findings indicate that real-time monitoring is underused, although early implementations indicate promising directions for future adaptive mental health systems.

#### Personalization and Intervention Strategies

Mood-based personalization was one of the most used defined methods; it was used in 4 studies [35,39,42,43]. Clinical stratification and risk profiling were used in 3 studies [22,26,27]. However, 2 studies [28,29] used AI-powered therapeutic interfaces, while 2 studies [40,41] were on behavior-based personalization. In addition, 2 studies [34,45] implemented treatment response prediction. Moreover, general personalization frameworks without dynamic adaptation were present in 2 studies [24,48]. These patterns indicate growing interest but highlight the need for more consistent and transparent personalization models.

#### AI Methodology and Implementation Challenges

The studies we reviewed used a number of different AI technologies, such as traditional machine learning approaches [22], including Random Forest and its variants like Boruta and Binary Mixed Model [23,24,35,36,40,44-47]. Moreover, we found ensemble approaches combining models like Naïve Bayes, decision trees, support vector machine, logistic regression, linear models, and voting regressors [27,28,38]. Furthermore, we saw several natural language processing techniques, including deep learning models such as recurrent neural networks, long short-term memory, gated recurrent unit, convolutional neural network, Efficiently Learning an Encoder that Classifies Token Replacements Accurately, and Bidirectional Encoder Representations from Transformers which were used for personalized recommendations [30,31,39,43-45].

Several studies reported key implementation challenges. These included issues with interpretability in deep learning models [29], data variability across sessions and algorithms [22]. Other issues were also found, such as chatbot systems' limited scope and privacy concerns [28,30]. Moreover, data imbalances related to antidepressant response also affected model performance [33].

#### Critical Influencing Factors

The reviewed studies identified a wide range of influencing factors that affect the efficacy of AI-driven personalized mental health interventions. Among the eligible studies, 3 studies [37,40,46] expressed concerns regarding treatment duration and adherence, and 3 others [34,35,38] reported technical problems, such as small datasets or feature limitations. Besides, 2 studies [29,41] showed concerns about privacy and autonomy (such as hesitancy to share data or fear of overprediction) while dissatisfaction with excessive personalization and monitoring emerged in the studies by Chen et al [42] and Alslaity et al [43]. Moreover, in 4 studies [22,25,27,33], demographic and psychosocial factors were highlighted. Other distinct factors included generalizability [44], mood tracking [39], sensor reliability [24], and personalized risk factors [26]. These results emphasize the need for future AI systems for personalized mental health to have a multifaceted design and more transparent reporting.

#### Target Groups and Conditions

Although some studies focused on clinically diagnosed groups [38-40] and young adults or adolescents [37,41], most studies [24,25,27,33-35,38,44,46,48] focused on adults between the ages of 18 and 65 years. Regional demographics were referenced by Kim et al [22] and Jensen et al [25], and a gender-specific focus surfaced in the study by Meinlschmidt et al [39]. The need for better reporting and broader inclusion in future research is highlighted by the fact that 8 studies [26,28-32,42,43] lacked accurate population data.

### RQ3: Explainability, Clinical Integration, and AI Decision Impact

#### Explainability Techniques

The reviewed studies used different approaches to explain AI. The majority of studies (21.4%) used tree-based and classical models [22,26,35,43,45,46], whereas 8 (28.6%) studies [23-25,28,31,32,36,47] did not report any explanation approach. Interpretability of deep learning was mentioned in 2 (7.1%) studies [29,49], along with NLP-based tools [30,42], feature selection techniques [27,34], model evaluation visualizations [38,39], and feature importance tools [33,37]. Three (10.7%) studies used hybrid or other methods [41,44,48], and 1 (3.6%) study reported explainability based on naive Bayes [40]. These findings highlight the need for more consistent and transparent AI systems in mental health by exposing unequal adoption of explainability practices.

#### Design and Implementation Challenges

The reviewed studies identified common deployment-related and technical issues in the design of AI systems for mental health. A notable issue was the lack of reporting in 14 (50.0%) studies [23-27,31,32,36,44-49]. However, concerns regarding the integration of devices and sensors were shown in 2 (7.1%) studies, and 4 (14.3%) studies brought up the subject of data variability and quality [22,33,34,37]. Two (7.1%) studies reported privacy and scope limitations, particularly in chatbot systems [28,30]. Other challenges included misclassification and generalization gaps [42], model overfitting [39], user engagement issues [40], and long-term trust erosion [43]. These findings show that more open and consistent reporting is needed to ensure the clinical adaptability of AI systems.

#### Trade-Offs Between Performance and Interpretability

The reviewed studies were markedly inconsistent with respect to the trade-off between explainability and AI model performance. Interpretability issues, particularly in deep learning models, were discovered in 4 (14.3%) studies [37,39,42,43]. Three (10.7%) studies raised broad concerns about finding a balance between clinical usability and accuracy [22,35,38]. Two (7.1%) studies reported high-performing but opaque models [33,34], and 2 (7.1%) more emphasized the conflict between transparency and personalization [40,41]. Interestingly, no discussion of this topic was reported in 17 (60.7%) studies [23-32,36,44-49]. These discrepancies highlight how explainable AI is necessary to improve clinical integration and trust.

#### Clinician-Centered Usability

Four studies [22,34,39,43] raised concerns regarding clinician acceptance and applicability, suggesting hesitancy to rely on AI systems without conclusive validation or clinical practice alignment. In 2 studies [35,37] that documented device dependency and relevance, wearable-specific data raised questions about clinical reliability. Lack of workflow integration [40-42], privacy and sensitivity restrictions [24,28], and restricted generalizability [45] were further difficulties. Only one study [38] specifically addressed explainability issues, and another [27] raised doubts about the effectiveness of the intervention. A significant gap exists in addressing real-world integration, as evidenced by the 13 (46.4%) studies [23,25,26,29-32,36,44,46-49] that did not identify any clinician-related concerns. These results highlight how crucial it is to build AI systems that promote clinician trust, usability, and practicality.

#### Impact on Decision-Making

In our review, studies showed different but promising effects on making clinical decisions. About 32.1% (9 out of 28) [22,26,27,29,33-35,43,45] agreed that depression should be diagnosed and its severity predicted. About 21.4% (6 out of 28) [23,37,39-41,46] were focused on giving personalized treatment recommendations. At the same time, 10.7% (3/28) studies [28,30,42] focused on chatbot-based or digital therapist roles, and 7.1% (2/28) studies [36,44] reported that patient-centered outcomes like planning daily activities and reducing symptoms had improved more effectively. The findings indicate that AI is becoming increasingly crucial in improving mental health care, but they also show that more real-world testing is needed.

### Transitioning From Systematic Findings to Framework Design

Building upon the findings from this systematic review, we identified persistent challenges within the realm of AI-driven mental health interventions. Specifically, the lack of adequate personalization, minimal explainability, and insufficient integration into established clinical workflows. These deficiencies are particularly significant in high-burden health care domains, such as AD/ADRD, where caregivers contend with both emotional distress and intricate informational demands. The insights we gathered from analyzing different studies demonstrated the importance of developing a more adaptive, transparent, and contextually aware AI framework. In the subsequent sections, we have used our findings to propose a dynamic personalization and explainable decision-making enabled human-AI collaborative system for caregivers of individuals with ADRD.

## Discussion

### Principal Findings

In this review, we analyzed 28 papers on personalized AI-powered mental health treatment strategies by examining the types of data used to detect early warning signs of mental disorders. We also explored how AI-enabled systems deliver real-time monitoring and dynamically personalize treatment plans based on individual user behavior and engagement patterns. Moreover, we identified the challenges associated with designing and validating explainable AI models. We also discussed the limitations of the reviewed studies following our RQs, to identify key gaps and opportunities for future work, as outlined below.

### RQ1: Limitations in Data Sources and Processing Practices

**Figure 4:**
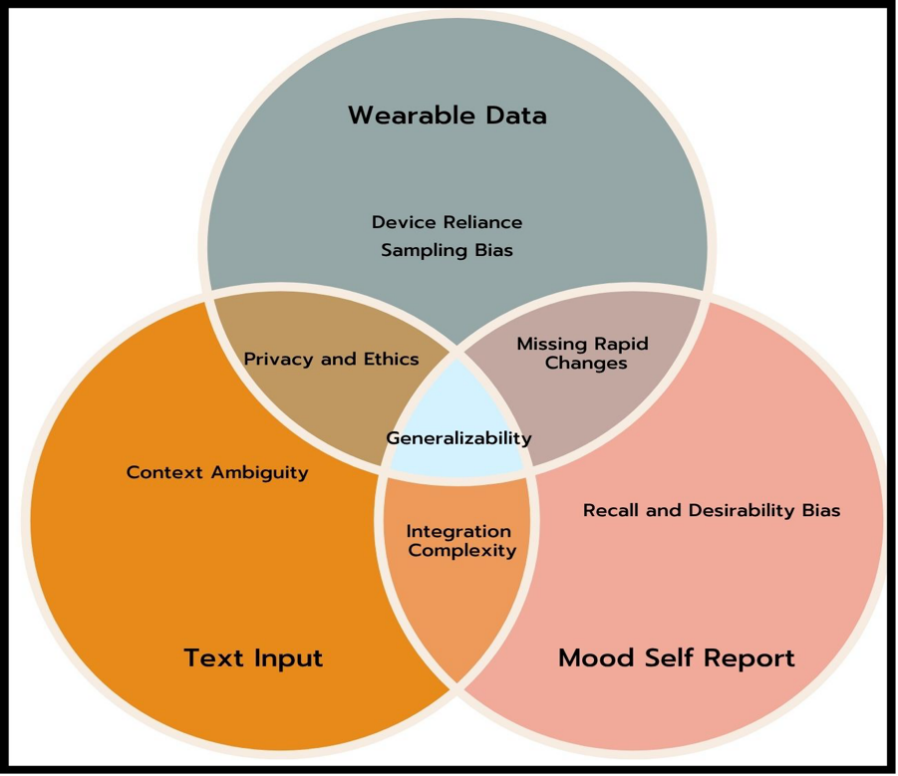
Key limitations across data types.

When designing personalized mental health intervention tools, there is no “one-size-fits-all” solution, particularly when it comes to selecting and tailoring input data sources [72]. In our review, it was visible that different studies used different approaches. In our review, though we found a large number of studies used self-reported questionnaires to get information about mood and symptoms [31,34,35,38,39,48], there are some major issues associated with this type of input. For instance, Stone et al [73] highlighted some of the major issues in explaining EMA in behavioral medicine. According to the author, a major issue with traditional self-reported questionnaires is recall bias, which means that people might forget, underreport, or misrepresent how they felt or what symptoms they had in the past. Another issue is social desirability bias, which happens when people change their answers to make them seem more acceptable or favorable [74]. Furthermore, some studies used NLP-derived textual features [31,32,42] to include detailed insights. However, these studies might face issues including contextual ambiguity [75], domain adaptation [76], and ethical concerns related to privacy and explainability [77,78]. The key limitations of the key data types are shown together in Figure 4.

In addition to these choices for collecting data, our review also found that reporting practices were not very clear or consistent. There was a significant gap with enough details about the steps for preprocessing and integrating data. For example, only a few of the 6 (21.4%) studies that used surveys and interviews [31,34,35,38,39,48] clearly explained how these were processed or combined with other types of data. These gaps make it hard to see important methodological choices about data cleaning, normalization, and integration that can have a significant impact on AI performance [79,80]. Last, we found that methodological integration and performance evaluation were underexplored. For instance, only 2 studies [40,41] looked at how to combine different types of data, like clinical records with smartphone-based sensing or wearable-derived physiological signals. In the same way, only 2 (7.1%) studies [23,29] looked at model performance indicators like accuracy and prediction.

### RQ2: Limitations in Personalization Strategies and Real-Time Monitoring

In the last several years, mental health systems have been increasingly moving toward personalization based on the idea that no 2 people deal with mental health issues in the same manner [72]. In personalization, real-time monitoring is one of the crucial variables. Traditional static AI mental health care approaches rely on retrospective or infrequent assessments that fail to capture dynamic, context-dependent symptom changes. Real-time monitoring solves this problem by gathering continuous, real-world data on mood and behavior [81]. Therefore, real-time monitoring tools enable digital mental health technologies that are aware of the environment to provide more flexible and effective treatment [78]. However, in our review, we found that even though real-time monitoring tools are essential, they were not widely used or used consistently in our studies. Instead, many studies used static or scheduled assessments that are incapable of detecting quick mood changes or environmental triggers. For example, studies [33,34] used retrospective mood questionnaires that were given weekly or at clinic visits, which are unable to inform timely, personalized interventions.

In our review, different personalization methods were found in mental health AI interventions, such as mood-based personalization [35,39,42,43], clinical stratification or risk profiling [34,40,41,45], and general personalization frameworks without dynamic adaptation [24,48]. However, all these personalization methods have some drawbacks. For instance, mood-based personalization often uses self-reported ratings from a single source that fails to consider evolving, contextual, or physiological factors that are important for adaptive mental health support [82,83]. In addition, clinical stratification and risk profiling often use static baseline assessments that disregard considering behavior or physiology, which makes adaptive prediction less accurate [84,85]. General personalization frameworks, on the other hand, use fixed rules that cannot change when users do, which makes them less relevant and engaging [86]. These limitations indicate significant gaps where multimodal data integration might be beneficial. Multimodal data integration can provide a comprehensive view of mental health states by combining different types of data, like physiological signals from wearables, self-reported mood ratings, and text written by users.

### RQ3: Limitations in Explainability, Generalizability, and Clinical Integration

Explainability is one of the main ingredients of personalization because it helps users understand and trust AI-driven decisions. For instance, in the study by Kaur et al [87], participants used a bird identification system with 4 different explainable AI methods, and reported that explanations helped them calibrate their trust in the AI’s output and improve their own identification skills. This finding illustrates how meaningful explanations can increase user confidence and support effective use of AI systems, a principle that is equally important in sensitive contexts like mental health care. In our review, we found that while many studies used tree-based and classical models for inherent interpretability [22,26,35,43,45,46], others used deep learning interpretability [29,49], NLP-based tools [30,42], feature selection [27,34], model evaluation visualizations [38,39], feature importance tools [33,37], hybrid methods [41,44,48], and naive Bayes approaches [40]. However, several studies did not report any explainability approach at all [23-25,28,31,32,36,47]. Therefore, this highlights the ongoing need for more consistent and thoughtful use of explainability techniques in mental health AI systems.

Our review revealed that few studies adequately addressed the issue of generalizability. The study by Webb et al [45] specifically highlighted that models trained on limited or homogeneous data may demonstrate suboptimal performance across varied demographic, cultural, or clinical contexts, thereby emphasizing the risk of inequitable outcomes in real-world mental health care. Besides, since this limitation is rooted in the diversity of people whose data is used, inputs such as wearables, mood-based self-reports, and text data alone do not inherently guarantee broader generalizability [88]. At the same time, another critical challenge in developing AI for mental health is balancing predictive performance with interpretability. Several studies in our analysis chose high-performing but black-box models [33,34] without sufficiently addressing how their lack of transparency may undermine clinician confidence or safe implementation. Another issue that is quite common in AI interventions related to mental health is the trade-off between model accuracy and practical usability. For instance, a limited number of studies [22,35,38] clearly addressed the trade-off between model accuracy and practical usability, underscoring a deficiency in design thinking crucial for real-world implementation. However, users often choose systems that are somewhat less accurate but more understandable, since they enhance comprehension, trust calibration, and informed decision-making [87]. Therefore, carefully addressing this trade-off is essential to guarantee that AI systems provide clinically significant help while maintaining transparency and acceptability for both practitioners and patients.

### Proposed Conceptual Framework

#### Overview

In this study, we are proposing a conceptual framework for personalized mental health care specifically for caregivers of patients with AD/ADRD. This framework is based on the findings of our systematic review of AI-powered personalized mental health treatment or care interventions.

**Figure 5:**
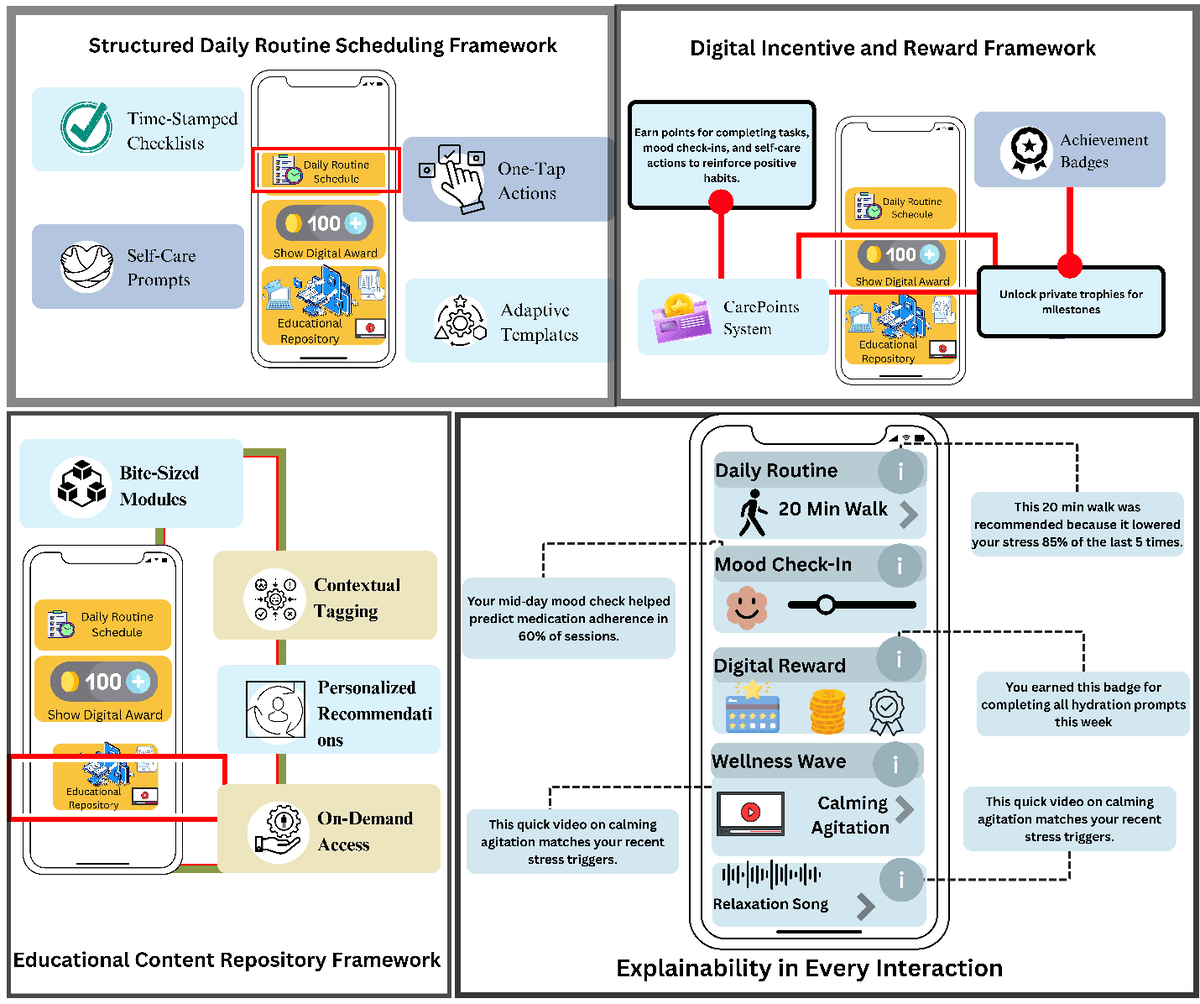
Key features of the proposed framework.

**Figure 6:**
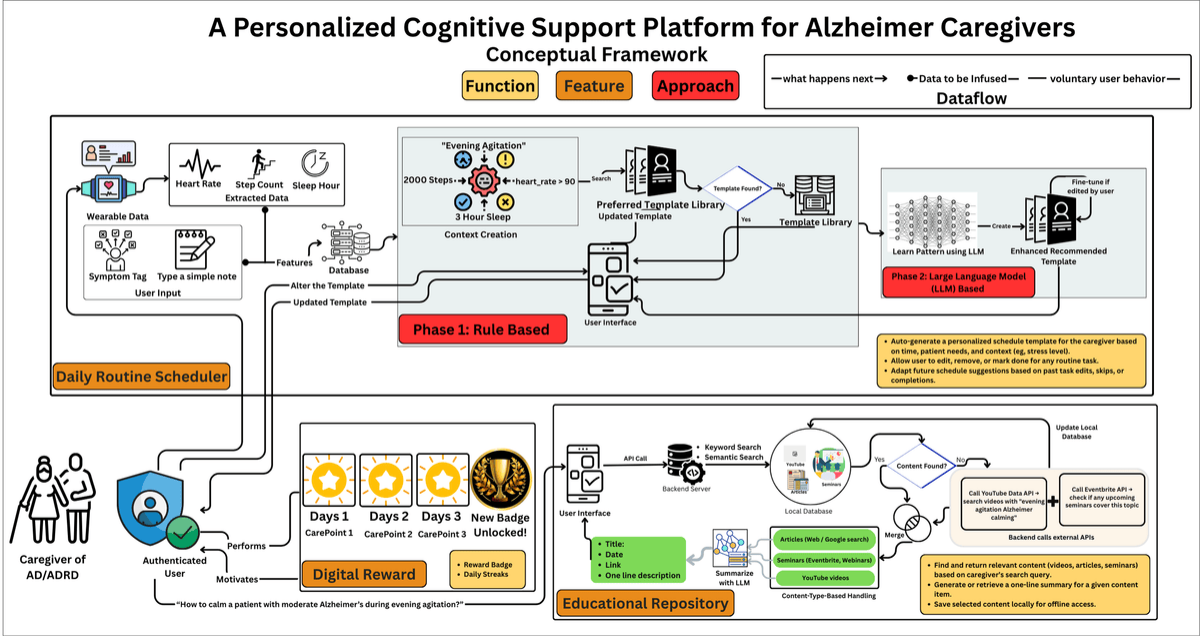
Overview of our proposed framework. AD/ADRD: Alzheimer disease and related dementias; API: Application Programming Interface.

In our conceptual framework, we propose integrating wearable-derived physiological signals, mood-based self-reports, and free-text entries describing users’ feelings to capture multidimensional, context-rich data that supports personalized mental health interventions. This approach emerges as complementary based on the evidence in Table 1. By following EMA [73], the framework is designed to take the user input multiple times daily. These multimodal data, followed by EMA, will solve the issues that we found in our review, such as recall bias and social desirability bias. However, this proposed framework has primarily 3 main features, such as a daily routine scheduler, a digital reward system, and an educational repository, as shown in Figure 5. This framework represents a significant advancement over prior approaches, which have primarily concentrated on developing conceptual frameworks or taxonomies within the field. The whole overview of our framework is shown in Figure 6.

#### Daily Routine Scheduler

A large portion of the routine of caregivers of individuals with AD/ADRD consists of the patient’s daily activities. Therefore, if caregivers are given a daily routine scheduler that combines the necessary needs and schedules of patients with AD/ADRD, it will be more beneficial for them. Keeping that in mind, the daily routine scheduler feature is composed of some subfeatures. First, there will be time‑stamped checklists for activities of daily living of patients with AD/ADRD, such as medications, meals, hygiene, and exercises. This will reduce the cognitive load of caregivers and ensure the attendance of critical things such as medication dosage.

Second, by enhancing the intrinsic motivation of self-determination theory [89], the system will have premade templates according to the caregiver’s input. According to Jin et al [89], allowing users to modify certain things in the system and receiving encouragement while doing so (Autonomy) is an important factor. Therefore, in our framework, a premade template will be given to the caregivers, and they will have the autonomy of alterations according to their preferences. When the caregivers make any changes to the templates, the system will learn from the changes, and it will provide a better template next time by using those learnings. Additionally, the system will provide suggestions that adjust task durations and prompts over time according to mild, moderate, and severe Alzheimer. This will match the changing abilities without the caregiver manually building schedules.

Third, to support the caregiver’s wellbeing, there will be self-care routine which will have scheduled self‑care breaks, hydration, short exercises, mood check‑in prompts that feed a private mental‑health log, which combinedly promotes caregiver health and flags early burnout. Through the mood checking prompts, the app will suggest some low-burden, context-aware prompts, such as a one‑tap breathing exercise, a 5‑minute stretch video, or a brief “Pause and hydrate” reminder right on the home screen, which shows the relatedness [89]. Therefore, together, these features turn the scheduler into a smart, caregiver‑centered system that balances patient care, caregiver’s health, and easy coordination with the wider support network.

#### Digital Incentive and Reward Framework

According to Jin et al [89], the user needs a sense of achievement to be motivated using a system, which is called competence. To ensure competence, the framework provides a feature of getting digital rewards, which will turn routine duties and self-care into incremental accomplishments, countering burnout by providing immediate, tangible affirmation. Whenever a caregiver completes a scheduled task, such as achieving milestones by logging mood checking, keeping a certain streak, 30-minute walks for 7 days, etc, a digital reward will be provided, which they might show as their progress to social media to encourage others.

#### Digital Educational Content Repository Framework

Despite having formal caregivers, family members are often involved in the care of patients with AD/ADRD as informal caregivers. Most of them do not have the proper training and knowledge to handle patients with proper care. Besides, their busy schedules do not allow them to get proper training or even to acquire knowledge from different sources. In that case, our app will help them immensely. To help them get the knowledge of patients with AD/ADRD–related articles or seminars, the app will provide them with bite-sized knowledge, which will be easy to adopt and time-efficient. If they want to know more about a specific article, they can go to the link by simply clicking on the bite-sized learning and get the full article. Moreover, our system will provide all related information, such as seminars, articles, and other educational sources, together for a better learning experience, which will decrease the workload of searching for necessary information in distinct locations.

### Future Directions

From the findings of the review, we found that the existing personalized mental health–related AI approaches have significant shortcomings in terms of explainability, transparency, and usability testing. In that case, our proposed framework directly addresses these deficiencies by furnishing a clear, user‑specific rationale for every recommendation—for example, a prompt suggesting a 20‑minute walk is justified by noting that the caregiver previously reported this activity as effective during periods of elevated stress. By consistently articulating the reasoning behind each action, the system enhances transparency, cultivates user trust, and supports sustained engagement.

The development of this conceptual framework presents profound opportunities for future work. Further research might incorporate the technology acceptance model to evaluate the adoption and usability of the proposed system. This will help to understand how users will accept and use this technology, focusing on 2 primary constructs such as perceived usefulness and perceived ease of use. Besides, according to Kushniruk and Patel [90], usability testing enhances generalizability by uncovering obstacles and design deficiencies across many user groups and circumstances, which will overcome the limitations found in the review. Therefore, deploying the framework in real-world environments will allow us to assess its scalability, integration with institutional routines, and effectiveness in supporting professional caregivers alongside family members.

### Limitations

This study has several notable limitations. First, none of the included studies were about patients with AD/ADRD or targeted caregivers. The studies were more focused on AI-driven personalized mental health care among the general population. However, this also serves as one of the strengths of this study. By analyzing the global advancement in personalized AI mental health interventions, we will be able to apply the outcome of the extracted foundational principles, such as mood-based personalization and explainability, from the review. This allowed us to design a forward-looking framework that can be adapted and validated in future contexts specific to caregivers of individuals with AD/ADRD.

Second, the proposed framework has yet to be evaluated through broader lenses with external experts. Third, there is a profound need for real-world usability studies to assess how caregivers interact with the system, perceive its usefulness, and engage with its adaptive and emotional support features. This has been reserved as one of the future works that will help the system to be fine-tuned.

Fourth, despite the review focused on studies that implemented AI-based personalized mental health interventions, we also included several papers in which the authors proposed that their models could potentially support personalization in future applications. This also worked as a strength of this study, as this enabled us to capture emerging perspectives and early-stage innovations, which kept us updated about the future landscape of AI-driven mental health care.

Fifth, the framework is currently suitable for home-based settings. However, we aim to facilitate the framework in a facility-based care setting and incorporate it with clinical experts. Finally, the review was done with studies that were published between January 2020 and February 2025; yet, new studies are continuing to be published. Therefore, an updated review of AI-driven mental health care interventions may be warranted in the near future.

Despite having these limitations, our proposed framework, especially on personalized mental health research on caregivers of patients with AD/ADRD, serves as a technically robust and future-proof design. However, future work is needed to check user acceptance and tailor these findings through caregiver-specific research.

## Appendix

Multimedia Appendix 1 PRISMA checklist.

Multimedia Appendix 2 Search query.

Multimedia Appendix 3 Detailed data sources, feature extraction, and processing methods.

Multimedia Appendix 4 Detailed personalization strategies and real-time AI-driven monitoring.

Multimedia Appendix 5 Detailed explainability, clinical Integration, and impact of artificial intelligence–mediated decisions.

## Abbreviations

AD/ADRD: Alzheimer disease and related dementias
AI: artificial intelligence
EMA: ecological momentary assessment
NLP: natural language processing
PRISMA: Preferred Reporting Items for Systematic Reviews and Meta-Analyses
RQ: research question
