# Supplementary material for: AI-Driven Mental Health Support for Caregivers of Individuals With Alzheimer Disease: Systematic Literature Review and Development of a Conceptual Framework
Source: JMIR Ment Health. 2026 Mar 6;13:e79973. doi: 10.2196/79973 (PMC13005065; doi:10.2196/79973)
Supplement: Multimedia Appendix 2 [file mental_v13i1e79973_app2.pdf]

PubMed:

1. ("personalized treatment" OR "precision medicine" OR "individualized treatment") AND ("mental health" OR "psychiatric disorders" OR "depression" OR "anxiety" OR "bipolar disorder") AND ("mHealth" OR "mobile health" OR "digital health" OR "eHealth" OR "app" OR "application") AND ("artificial intelligence" OR "AI" OR "machine learning" OR "deep learning")
   (Total 8 paper)
2. ("personalized treatment" OR "precision medicine" OR "individualized treatment") AND ("mental health" OR "psychiatric disorders" OR "depression" OR "anxiety" OR "bipolar disorder") AND ("mHealth" OR "mobile health" OR "digital health" OR "eHealth") AND ("artificial intelligence" OR "AI" OR "machine learning" OR "deep learning")
   (Total 15 papers)

Google Scholar:

1. ("personalized treatment" OR "precision medicine" OR "individualized treatment") AND ("mental health" OR "psychiatric disorders" OR "depression" OR "anxiety" OR "bipolar disorder") AND ("mHealth" OR "mobile health" OR "digital health" OR "eHealth" OR "app" OR "application") AND ("artificial intelligence" OR "AI" OR "machine learning" OR "deep learning")

(Total 15 Papers)

2. (intitle:"personalized mental health" OR intitle:"precision psychiatry" OR intitle:"individualized therapy")

AND ("mental health intervention" OR "digital therapeutics" OR "psychiatric care")

AND ("mobile applications" OR "telemedicine" OR "mHealth apps" OR "eHealth platforms")

AND ("artificial intelligence" OR "machine learning" OR "deep learning" OR "predictive analytics")

(Total 6 papers)

IEEE Xplore:

1. (("personalized treatment" OR "precision psychiatry") AND ("mental health" OR "depression" OR "bipolar disorder") AND ("mHealth" OR "digital health") AND ("AI" OR "machine learning"))
   (Total 1 Paper)

2. ("All Metadata":personalized treatment OR "All Metadata":precision psychiatry OR "All Metadata":individualized therapy) AND ("All Metadata":Mental Health OR "All Metadata":depression OR "All Metadata":bipolar disorder OR "All Metadata":ANXIETY)
   (Total 10 Papers)

Scopus:

1. TITLE-ABS-KEY (mental* AND ai AND personalized*) TITLE-ABS-KEY ("personalized treatment" OR "precision psychiatry" OR "individualized therapy") AND TITLE-ABS-KEY ("mental health" OR "depression" OR "anxiety" OR "stress" OR "bipolar disorder") AND TITLE-ABS-KEY ("artificial intelligence" OR "AI" OR "machine learning") AND PUBYEAR > 2020 AND PUBYEAR < 2025
   (Total 4 papers)
2. TITLE-ABS-KEY (mental* AND ai AND personalised*) TITLE-ABS-KEY ("personalized treatment" OR "precision psychiatry" OR "individualized therapy") AND TITLE-ABS-KEY ("mental health" OR "depression" OR "anxiety" OR "stress" OR "bipolar disorder") AND TITLE-ABS-KEY ("artificial intelligence" OR "AI" OR "machine learning") AND PUBYEAR > 2020 AND PUBYEAR < 2025
   (Total 9 papers)

ScienceDirect:

1. mental health and (AI OR Machine Learning) AND (personalized OR precision)
   (Total 5 papers)
