# Supplementary material for: AI-Driven Mental Health Support for Caregivers of Individuals With Alzheimer Disease: Systematic Literature Review and Development of a Conceptual Framework
Source: JMIR Ment Health. 2026 Mar 6;13:e79973. doi: 10.2196/79973 (PMC13005065; doi:10.2196/79973)
Supplement: Multimedia Appendix 3 [file mental_v13i1e79973_app3.pdf]

Table 1. Detailed Data sources, feature extraction, and processing methods.

Study Type	Data Source	Collection Method	Feature Extracted	Pre-processing Techniques	Data Integration	AI Model Used	Model Challenges
Experimental Study [1]	Participants from Clinical Trial	Participants and patients from the Research on Asian Psychotropic Prescription Patterns for Anti-Depressants (REAP-AD)	Severe Depression, Age	Did not mention and/or not found	Combining Data from Multiple Demographics	Predicting Psychotic Disorders	Improvement in sensitivity
Experimental Study [2]	Participants from Clinical Trials, Data from previously treated patients	A sample of psychotherapy patients from an Outpatient Clinic	True errors for accuracy	Utilization of various patients such as The Hopkins-Symptom Checklist, the Berne Post-Session Report, and the Boruta Algorithm	Not applicable with one sample of patients	Predicting psychotherapy treatment outcomes and associations	Limited use to therapists, Accuracy of the model, different prediction selection algorithms
Randomized and Integrated Study [3]	Data from Participants in Clinical Trial	N/A	Environmental Biomarkers	N/A	N/A	Treatment for Individuals with Major Depressive Disorder	N/A
Cohort Study [4]	Data from Participants in Clinical Trial	Multi-modal investigative program	Environmental Biomarkers	Pre-Examination across six outpatient clinics within the capital region of Denmark	Integration of various data sets with different metrics	Identify biomarkers to predict treatment outcomes for patients with MDD	N/A
Meta-Analysis [5]	Data from participants in Clinical Trial	A Large and Varied Dataset consisting of individual level data.	Characteristics of beneficiaries, subgroups of beneficiaries	N/A	Combination of 26 Datasets containing a total of 1544 Participants	Using Machine Larning Algorithms for personalized cognitive training	Efficacy of the intervention models
Analysis [6]	Multimodal Data Set	The Multimodal Dataset of	Depression Severity, Personalize	Merging and Sorting of Multiple	Merging and Sorting of Multiple Sub	Using 5 Different Machine Learning	N/A

		the National Health and Nutrition Examination Surveys (NHANES)	d Risk Factors	Sub data sets into one	data sets into one	Algorithms to Predict Depression Severity and Personalized Risk Factors	
Analysis [7]	SAAC AI Mental Health Chatbot	Dataset was mentioned but not specifically	N/A	N/A	N/A	Using an AI mental Health chatbot to offer personalized treatment	Response Speed, Tailored, Responses, Accuracy, Privacy and Confidentiality
Literature Review [8]	Clinical Trial Databases, Hospital Records, Surveys, Biobanks, and electronic health records	Datasets collected from multiple sources such as Clinical Trial Databases, Hospital Records, Surveys, Etc.	Accuracy of the models	Data Cleaning, Identification, and rectification of any missing values	N/A	Using various Deep Learning Models end evaluating how effective they are in personalized treatment for anxiety disorders	N/A
Literature Review [9]	Kaggle, GitHub, and Reddit	Kaggle and GitHub Datasets were used, along with Data being gathered from Reddit	Chatbot Features	N/A	N/A	Utilizing Natural Language and Deep Learning Processes to create an AI-Based mental health care chatbot	Accuracy, Tailored Responses, Privacy
General description how AI can be helpful for improving personalized treatment plan [10]	Comprehensive surveys and interviews	Surveys, interviews	User's feelings, expressions, sentiment	NLP, sentiment analysis	N/A	None	N/A
General description how AI can be helpful for	N/A	N/A	user intent recognition, entity extraction, sentiment analysis		N/A	None	N/A

improving personalized treatment plan [11]							
Experimental Study [12]	Outpatients diagnosed with non-melancholic depression	N/A	Depression type, Age, treatment-resistant depression history, comorbid psychiatric or medical conditions, BMI, HAM-D baseline scores	Noise reduction (Encoding, Standardization, Normalization)	N/A	Gradient Boosting Classifier, Feature importance analysis	Class imbalance, prediction challenges
Experimental Study [13]	Meru health (healthcare provider via smartphone)	Meru health app: from participants self-disclosures and questionnaires presented on the app or data entered by care coordinator on the app	Sex, Age, Referral, payment, Motivation, medication PHQ-9 baseline, GAD-7 baselines, WPAI, burnout score, history of trauma etc.,	Dichotomizing variables, scikit-learn	N/A	Random Forest Classifier	Lower predictive performance due to missing data and biased variable importance, differences between test set and train set, lacks realistic baseline
Observational study [14]	Wearable devices, self-report questionnaire	Fitbit Data, questionnaire: PHQ-8 and ASRM	Sleep, activity, heart rate, step count, depression symptom severity (PHQ-8), manic symptom severity (ASRM)	Limited filtering criteria, random forest imputation method on Fitbit data	N/A	BiMM (Binary Mixed Model Forest)	Highly dependent on end user Fitbit compliance
General description how AI can be	N/A	N/A	N/A	N/A	N/A	N/A	N/A

helpful for improving personalized treatment plan [15]							
Experimental Study [16]	BrainE app, Wearable devices (Samsung Galaxy Wristwatch	PHQ-9 scale (score > 9), suicidal behavior (Columbian Suicide Severity Rating scale), EMA (Ecological momentary assessments), Neurocognitive assays on EEG (Electroencephalography) platform, cognitive assessment games	Sleep, Physical activity, stress metrics, inhibitory control, interference processing, working memory, emotion bias, internal attention, reward processing, diet reporting	Data imputation, standardization and regularization, preprocessing pipeline object (to avoid leakage statistics from test into training model in CV)	Combining Data from Multiple Platforms	(Each participant has own personalized model) Elastic net, Radon Forest, Gradient Booster, Support vector, Poisson Regressor, Ada Boost and then Voting Regressor, SHAP (SHapley Additive exPlanations)	low sample size (data from 14 participants),
Experimental Study [17]	Clinical trials (from 6 clinical trials of pharmacological treatment for MDD: COMED, STAR*D, REVAMP, EMBARC, IRL-GREY, SUN☺D)	Patient demographics, questionnaires	Age, Sex, Race/ Ethnicity, intensity of various depression symptoms recorded in questionnaires	Data transformation	Merging different datasets	Differential Prototype Neural Network	Loss of features due to inconsistency in datasets, limited the possible differentiation between subgroups, lack of data from different treatment modalities
Experimental study [18]	Smartphone (data were collected within randomized trial)	Multidimensional mood changes questionnaire (before and after	Marital Status, Highest degree, size of household, smartphone	Data imputation	N/A	Random forest	Tension between accuracy and predictability as the model does not layout firm conclusions on exact contribution

		psychotherapeutic micro intervention, EFF survey)	usage, age, response to micro intervention				of each factor to model's predictability
Feasibility Study [19]	Smartphone-based system (MUBS)	User interaction (17 user) with the mobile app	User activity data (planned & completed activities), mood tracking, engagement	Activity categorization, removal of incomplete/missing data, Feature extraction from logs	N/A	Multinomial Naïve Bayes (for personalized activity recommendations) LME model (to examine app usage)	Contextual awareness (e.g., suggesting activities irrelevant to user context)
Randomized placebo study on the "Foundations" mental health app. [20]	Foundations mental health app user self-reports and mobile sensor data.	User engagement tracking via app interactions.	Personality traits and user engagement behavior.	Data normalization and aggregation.	N/A	AI-based recommendation system to enhance user experience.	1. Balancing user autonomy with personalized AI recommendations posed a challenge as users preferred more guidance but engaged more with autonomy.<br>2. Ensuring the effectiveness of personalization without creating a paradox of choice, where too many options reduce user engagement.<br>3. Ethical concerns related to AI-driven mental health recommendations, particularly around paternalistic design choices versus respecting user autonomy.
AI-infused system study in online mental health communities. [21]	Online mental health community uses text data and AI-generated responses.	Sentiment analysis on user-generated text in support forums.	Emotional support phrases and user reactions.	NLP for emotional content analysis.	N/A	AI-assisted emotional support system to facilitate peer engagement.	1. Bias in AI-generated emotional responses, as automated support messages might misinterpret or fail to fully capture human emotions.<br>2. The challenge of

								making AI responses empathetic and personalized while ensuring they are contextually relevant and do not create unintended distress.<br>3. Ensuring the AI system supports rather than replaces human interaction in online communities, maintaining trust in peer-support environments.
Longitudinal study analyzing Moodie mental health app data over two years. [22]	Self-reported mood tracking and activity logs from Moodie.	Longitudinal mood tracking and seasonal correlation.	Mood variations linked with daily activities.	Seasonal data correlation and trend analysis.	N/A	Machine learning for mood prediction and behavioral trend analysis.		1. User engagement fluctuated over time, particularly due to seasonal variations affecting mood-tracking behavior.<br>2. Data privacy concerns emerged due to the continuous logging of user moods and behaviors, requiring a balance between personalization and user consent.<br>3. The challenge of making AI-driven mood predictions accurate without overfitting to seasonal trends or missing critical contextual factors in user mood shifts.
Analysis [23]	Genetic Profiles, fmri scans, Medical Records, Big healthcare data	Data was collected, analyzed, and filtered using various machine	N/A	N/A	Machine Learning Algorithms filtering and analyzing data.	Using AI and big healthcare data to give accurate and individualized mental health treatment		N/A

		learning algorithms					
Randomized Controlled Trial [24]	Employees from Wisconsin School Districts	Employees were recruited via email.	N/A	N/A	N/A	Using an AI-Based Mobile App to see who benefits from app-based mediation training	Exclusion of other characteristics and relevant variables
Experimental study [25]	Adult outpatients with MDD from a mental health care center in Netherlands	weekly treatment based on CT (cognitive therapy), IPT (interpersonal psychotherapy)	Depression, demographics, psychological distress, general functioning, psychological processes, life and family history	Correlation matric was used to prevent multicollinearity		Random forest algorithm	Model generalization to new samples, populations, and treatment setting in unknown
Evaluation of different ML models [26]	N/A	N/A	N/A	N/A	N/A	N/A	N/A
Experimental study [27]	De-identified clinical data of adults suffering from common mental disorders	questionnaire	Demographic variables, psychometric questionnaire, K10 or PSS	data transformation using one-hot encoding, standardize numeric scores	N/A	Braive's system	None mentioned
Literature study [28]	Social media	subreddit	Titles, posts and userIDs	Eliminate punctuations and white spaces from posts, filter stop words	N/A	Model designed using BERT, GRU, CNN	None mentioned

References:

1. Kim K, Ryu JI, Lee BJ, et al. A machine-learning-algorithm-based prediction model for psychotic symptoms in patients with depressive disorder. J Pers Med. 2022;12(8):1218. doi:10.3390/jpm12081218

2. Rubel JA, Zilcha-Mano S, Giesemann J, et al. Predicting personalized process-outcome associations in psychotherapy using machine learning approaches: A demonstration. Psychother Res. 2020;30(3):300–309. doi:10.1080/10503307.2019.1597994
3. Monaco F, Vignapiano A, Piacente M, et al. Innova4Health: an integrated approach for prevention of recurrence and personalized treatment of major depressive disorder. Front Artif Intell. 2024;7:1366055. doi:10.3389/frai.2024.1366055
4. Jensen KHR, Dam VH, Ganz M, et al. Deep phenotyping towards precision psychiatry of first-episode depression: the Brain Drugs-Depression cohort. BMC Psychiatry. 2023. doi:10.1186/s12888-023-04618-x
5. Amirhosseini MH, Ayodele AL, Karami A. Prediction of depression severity and personalised risk factors using machine learning on multimodal data. In: 2024 IEEE 12th International Conference on Intelligent Systems (IS); 2024; Varna, Bulgaria. p. 1-7. doi:10.1109/IS61756.2024.10705185
6. Shani R, Tal S, Derakshan N, et al. Personalized cognitive training: protocol for individual-level meta-analysis implementing machine learning methods. J Psychiatr Res. 2021;138:342–348. doi:10.1016/j.jpsychires.2021.03.043
7. J R, Vijayaraghavan A, K R A, et al. AI powered chatbot for mental health treatment. In: 2024 First International Conference on Technological Innovations and Advance Computing (TIACOMP); 2024; Bali, Indonesia. p. 168–172. doi:10.1109/TIACOMP64125.2024.00037
8. Pandey S, Sharma S, Wazir S. Mental healthcare chatbot based on natural language processing and deep learning approaches: Ted the therapist. Int J Inf Technol. 2022;14:3757–3766. doi:10.1007/s41870-022-00999-6
9. Ravichand M, Singh J, Shelke NA, et al. Evaluating the efficacy of deep learning models in personalizing treatment for anxiety disorders. In: 2024 4th International Conference on Intelligent Technologies (CONIT); 2024; Bangalore, India. p. 1–6. doi:10.1109/CONIT61985.2024.10626514
10. R K, Priyanka S, P S, et al. AI-driven approaches to enhancing mental wellbeing and stress relief. In: 2025 International Conference on Multi-Agent Systems for Collaborative Intelligence (ICMSCI); 2025; Erode, India. p. 925–931. doi:10.1109/ICMSCI62561.2025.10894494
11. K N, J U. MediBot: healthcare assistant on mental health and well being. In: 2023 7th International Conference on Computation System and Information Technology for Sustainable Solutions (CSITSS); 2023; Bangalore, India. p. 1–5. doi:10.1109/CSITSS60515.2023.10334083
12. de Filippis R, Foysal AA. Advanced machine learning models for gender-specific antidepressant response prediction overcoming data imbalance for precision psychiatry. Sci Res. 2025. doi:10.4236/oalib.1112895

13. Hornstein S, Forman-Hoffman V, Nazander A, et al. Predicting therapy outcome in a digital mental health intervention for depression and anxiety: a machine learning approach. Digit Health. 2021;7. doi:10.1177/20552076211060659
14. 1 Lipschitz JM, Lin S, Saghafian S, et al. Digital phenotyping in bipolar disorder: using longitudinal Fitbit data and personalized machine learning to predict mood symptomatology. Acta Psychiatr Scand. 2025;151(3):434–447. doi:10.1111/acps.13765
15. Eid MM, Yundong W, Benneh Mensah G, et al. Treating psychological depression utilising artificial intelligence: AI for precision medicine – focus on procedures. Mesopotam J Artif Intell Healthc. 2023;2023:76–81. doi:10.58496/MJAIH/2023/015
16. Shah RV, Grennan G, Zafar-Khan M, et al. Personalized machine learning of depressed mood using wearables. Transl Psychiatry. 2021;11:338. doi:10.1038/s41398-021-01445-0
17. Benrimoh D, Kleinerman A, Furukawa TA, et al. Towards outcome-driven patient subgroups: a machine learning analysis across six depression treatment studies. Am J Geriatr Psychiatry. 2024;32(3):280–292. doi:10.1016/j.jagp.2023.09.009
18. Meinlschmidt G, Tegethoff M, Belardi A, et al. Personalized prediction of smartphone-based psychotherapeutic micro-intervention success using machine learning. J Affect Disord. 2020;264:430–437. doi:10.1016/j.jad.2019.11.071
19. Doe J, Smith A, Lee B. MUBS: a personalized recommender system for behavioral activation in mental health. In: Proceedings of the 2020 CHI Conference on Human Factors in Computing Systems; 2020. p. 1–12. doi:10.1145/3313831.3376795
20. Johnson K, Williams M, Zhao Y. Personalized recommendations in mental health apps: the impact of autonomy and data sharing. In: Proceedings of the 2021 CHI Conference on Human Factors in Computing Systems; 2021. p. 1–13. doi:10.1145/3411764.3445678
21. Chen L, Kim H, Patel R. Exploring the effects of AI-assisted emotional support processes in online mental health communities. In: Extended Abstracts of the 2022 CHI Conference on Human Factors in Computing Systems; 2022. p. 1–8. doi:10.1145/3491101.3503658
22. Alslaity A, Chan G, Orji R, et al. Insights from longitudinal evaluation of Moodie mental health app. In: Extended Abstracts of the 2022 CHI Conference on Human Factors in Computing Systems (CHI EA '22); 2022. Article 308. p. 1–8. doi:10.1145/3491101.3519851
23. Sharma MK, Nachappa MN, Kumar R. Personalized treatment recommendations for mental health disorders using AI and big healthcare data. In: 2023 IEEE International Conference on ICT in Business Industry & Government (ICTBIG); 2023; Indore, India. p. 1–6. doi:10.1109/ICTBIG59752.2023.10455991
24. Webb CA, Hirshberg MJ, Davidson RJ, et al. Personalized prediction of response to smartphone-delivered meditation training: randomized controlled trial. J Med Internet Res. 2022;24(11):e41566. doi:10.2196/41566

25. van Bronswijk SC, DeRubeis RJ, Lemmens LHJM, et al. Precision medicine for long-term depression outcomes using the Personalized Advantage Index approach: cognitive therapy or interpersonal psychotherapy? *Psychol Med*. 2021;51(2):279–289. doi:10.1017/S0033291719003192
26. Ramzan HA, Abdulah F, Ahmad M, et al. AI-driven personalization of e-therapy interventions for anxiety, stress, and depression. In: *2024 18th International Conference on Open Source Systems and Technologies (ICOSST)*; 2024; Lahore, Pakistan. p. 1–6. doi:10.1109/ICOSST64562.2024.10871158
27. Schmidt F, Hammerfald K, Jahren HH, et al. Using machine learning to recommend personalized modular treatments for common mental health disorders. In: 2023 IEEE International Conference on Digital Health (ICDH); 2023; Chicago, IL, USA. p. 150–157. doi:10.1109/ICDH60066.2023.00030
28. Pushpa G, Chaitra M, Kolur LP, et al. An advanced AI framework for mental health diagnostics using bidirectional encoder representations from transformers with gated recurrent units and convolutional neural networks. *Ing Sci Int J Inf Syst Intell*. 2025;30(1):213–220. doi:10.18280/isi.300118
