# Supplementary material for: AI-Driven Mental Health Support for Caregivers of Individuals With Alzheimer Disease: Systematic Literature Review and Development of a Conceptual Framework
Source: JMIR Ment Health. 2026 Mar 6;13:e79973. doi: 10.2196/79973 (PMC13005065; doi:10.2196/79973)
Supplement: Multimedia Appendix 4 [file mental_v13i1e79973_app4.pdf]

Table 2. Detailed personalization strategies and real-time AI-driven monitoring

Study Type & Data Source	AI Methodology & Approach	Real-Time Monitoring Scope	Personalization Strategy & Intervention Model	Critical Influencing Factors	Challenges in AI Implementation	Target Population & User Group	Associated Mental Health Conditions
Experimental Study, Data From Participants from Clinical Trial [1]	Machine-Learning Prediction Model, Random Forest Model	N/A	A Machine-Learning-Algorithm-Based Prediction Model to identify psychotic symptoms	Age, Degrees of Depression	Improvement in sensitivity	Various Demographics from Asia	Depressive Disorders and Symptoms
Experimental Study, Data from Participants from Clinical Trials, Data from previously treated patients [2]	Boruta Machine-Learning Algorithm, based on the Random Forest Model	N/A	Using the Boruta Machine Learning Algorithm to improve personalized psychotherapy treatment	N/A	Accuracy of the Algorithm, different predictor selection algorithms	One Sample from an Outpatient Clinic	Depression

Randomized and Integrated Study. Data from Participants in Clinical Trial [3]	N/A	Did not mention and/or not found	Technological Platform for personalized treatment with sensor integration, data processing, management, etc	Data accuracy, sensor accuracy, variation in treatment	Did not mention and/or not found	Individuals aged from 14-50	Major Depressive Disorders
Cohort Study, Data from Participants in Clinical Trials [4]	N/A	Informational Follow-ups and questionnaires in 6-month intervals after treatment initiation: 6, 12, and 18 months	Machine Learning Coupled with Statistical Models for Predicting Treatment Outcomes	Age, Treatment Duration	N/A	Adults Aged 18 –65 from the capital region of Denmark	Major Depressive Disorders
Analysis, Data from Multimodal Data Set [5]	A combination of 5 Different Machine Learning Algorithms, naïve bayes, decision tree, support vector machines, Random Forests, and Logistic Regression Algorithms	N/A	Using Machine Learning Algorithms to predict both Depression Severity and Personalized Risk Factors	Personalized Risk Factors	N/A	N/A	Depression Severity, Personalized Risk Factors

Meta-Analysis, Data from Participants in Clinical Trial [6]	Two Machine-Learning Based Models, Linear Model, and Ensemble Model.	N/A	Using Machine Learning to identify the effects of Cognitive Training, seeing which subgroups benefit more than others.	Age, Population, Pre-Post Assessment	The efficacy of the intervention models	Adults aged from 18-65, adults who are healthy and sub-clinical	Anxiety, Depression
Analysis [7]	User Interface and Sequential Model	N/A	Using SAAC to behave like a virtual therapist, and even eventually a pocket doctor	N/A	Response Speed, Accuracy, Privacy, Tailored Responses	N/A	Anxiety, Depression, Trauma

Literature Review, Data from various datasets [8]	Various Deep Learning approaches and natural language processing	N/A	Using the Chatbots as virtual therapists	What each chatbot offers in terms of treatment, privacy	N/A	N/A	Mental Disorder
Literature Review, Data from Various Databases and Health Records [9]	Using Three different deep learning models: Deep Reinforcement Learning, RNN, LSTM	N/A	Using the Deep Learning Models and seeing the efficacy of them in clinical therapy	N/A	N/A	N/A	Anxiety Disorders, Mental Health Disorders
General description how AI can be helpful for improving personalized treatment plan [10]	N/A	N/A	N/A	N/A	N/A	N/A	N/A
General description how AI can be helpful for improving personalized treatment plan [11]	N/A	N/A	N/A	N/A	N/A	N/A	N/A
Experimental study, Data from Outpatients diagnosed with non-melancholic depression [12]	Machine Learning based – Gradient Boosting Classifier, Feature importance analysis	Patient demographics, clinical metrics	ML Model capable of predicting gender-specific responses to anti-depressants	BMI, HAM-D scores (physical health and depression severity) rather than Gender	Class imbalance (where data disproportionately represents certain groups)	Individuals (aged 18-65) diagnosed with non-melancholic depression	Depression
Experimental, Data from Meru health app [13]	Machine Learning based – Random Forest Classifier	Questionnaire (social demographic and clinical variables)	ML Model capable of predicting response to treatment	Missing data	Interpretability, lacks realistic baseline, i.e., with the easily interpretable	Therapist supported, depression and anxiety participants	Depression and Anxiety

					data used for this approach, human clinicians can predict better than the algorithm		
Observational, Data from Wearables (Fitbit) [14]	ML based – BiMM (Binary Mixed Model Forest)	Real-time monitoring via Fitbit device	ML model capable of detecting mood symptomatology in bipolar disorder patients	More training data (would allow evaluating differences in prediction accuracy among diagnostic and demographic subgroups)	Prediction accuracy (due to differences in user Fitbit compliance in real world) fit bit data features are less precise	Adults (wearing Fitbit device)	Bipolar disorder I
General description [15]	N/A	N/A	N/A	N/A	N/A	N/A	Depression
Experimental study [16]	(Each participant has own personalized model) Elastic net, Radon Forest, Gradient Booster, Support vector, Poisson Regressor, Ada Boost and then Voting Regressor, SHAP (SHapley)	Monitoring via Samsung wristwatch	ML model capable of detecting depression	Medication adherence, Wearable device compliance	Variability in wearable device usage and sensor variabilities, App constraint	Young adults	Depression
Experimental Study, data from clinical trials [17]	Differential Prototype Neural Network	None	None	Feature limitation	Not yet ready for clinical implementation, variability in	Adults receiving pharmacological treatment	Major Depressive Disorder

					treatment options		
Experimental study, data from randomized trial [18]	Random Forest	None	ML model to detect mood changes	Mood change prediction from pre to post micro intervention	Lack of explainability (predictability differences between techniques), lack of understanding on exact contribution of factors	Male Adults (18 –65)	Not specified
Feasibility study (clinical + recruited users) [19]	Content-based probabilistic recommendation (Multinomial Naïve Bayes)	User self-reported activity tracking, passive data from mobile sensors (e.g., step count, weather)	Personalized activity recommendations using past activity history, mood-based suggestions	Activity tracking, patient engagement, clinical adherence	Lack of context-awareness in recommendations, challenge in engaging users consistently over time	Patients with depressive disorders (unipolar depression, bipolar disorder, OCD, anxiety)	Depression, anxiety, borderline personality disorder
Randomized placebo study with Foundations app data [20]	Naïve Bayes classifier for activity suggestions.	daily user activity, Real-time adherence tracking	AI-driven activity recommendations were tailored based on user preferences and stress levels.	paradox between autonomy and personalization, Participants hesitated to share mobile sensor data	randomized AI suggestions were perceived as personalized, influencing user behavior, Trust in AI-driven recommendations was lower when users felt their data privacy was at risk.	Young adults and working professionals, People hesitant to seek formal therapy but open to digital mental health solutions	Stress, Anxiety, Mild to Moderate Depression
AI-driven emotional support system in online mental health communities. [21]	ELECTRA (Efficiently Learning an Encoder that Classifies Token Replacements Accurately), KRWordRank for keyword extraction	The AI continuously monitored text-based peer support interactions in an online mental health community and provided empathetic response	AI-assisted emotional responses were personalized based on the detected sentiment and context of a user's post.	The risk of "over-personalization"	struggled to detect complex emotions like sarcasm, mixed feelings, or subtle distress signals, robotic responses = Empathy & Human-Like Interaction Deficit	N/A	Depression, Anxiety, Social Isolation, Emotional Dysregulation

Longitudinal study tracking user engagement in the Moodie app. [22]	Random Forest Classifier and XGBoost for mood prediction. LSTM for sentiment classification	Self-Reported Mood Tracking Over Two Years	AI dynamically adjusted recommendations based on user behavior, factoring in seasonal and daily variations in mood trends.	Users were concerned about continuous mood tracking, especially when combined with contextual data (e.g., location, activity logs).	struggled to differentiate between temporary mood fluctuations and long-term patterns, AI-driven reminders needed to evolve dynamically to maintain long-term interest, less transparency in data storation, analyzation and usage for AI recommendations	N/A	Mood Disorders, Seasonal Affective Disorder (SAD), Stress, Emotion Regulation, Mental Well-Being
Analysis, data from fmri scans, genetic profiles, and medical records [23]	Different Machine Learning Algorithms, Deep Learning for Diagnosis (DL-D), Collaborative Filtering for Treatment Recommendation (CF –TR), and Reinforcement Learning for Medication (RL- M)	N/A	Using algorithms to give accurate treatment for each individual patient.	Adaptability, and influence on public health	Accuracy performance of the various algorithms	N/A	Mental Health Disorders, Anxiety, Depression, Schizophrenia, Bipolar Disorder
Randomized Control Trail, Data from Wisconsin School District Employees [24]	Random Forest-Based imputation for missing data and two regressive models for outcome prediction	The trial was designed for the app to be used during a 4-week period.	Using Machine Learning Algorithms and models to predict who would benefit the most from mediation apps.	N/A	N/A	Adults who were fluent in English, had minimal knowledge of the App, and with Depressive Symptoms	Depression, Anxiety

Experimental study, data from randomized trial [25]	Random Forest	None	ML model to predict depression severity	Long term PAI vs post-treatment PAI indicated different treatment recommendations. One reason might be the time span	The degree to which the model can be applicable to new samples, populations, and treatment settings is unknown.	Adult population (18-65)	MDD (exclusion: bi-polar or highly chronic depression and high acute suicide risk)
[26]	N/A	N/A	N/A	N/A	N/A	N/A	N/A
[27]	Braive's system	None	ML model to provide treatment recommendations	Not specified	Not specified	adults	Treatment recommendations
Literature study [28]	BERT, GRU and CNN	None	Not specified	Not specified	Not specified	Not specified	Enhancing early identification, enabling focused interventions

References:

1. Kim K, Ryu JI, Lee BJ, et al. A machine-learning-algorithm-based prediction model for psychotic symptoms in patients with depressive disorder. J Pers Med. 2022;12(8):1218. doi:10.3390/jpm12081218
2. Rubel JA, Zilcha-Mano S, Giesemann J, et al. Predicting personalized process-outcome associations in psychotherapy using machine learning approaches: A demonstration. Psychother Res. 2020;30(3):300–309. doi:10.1080/10503307.2019.1597994
3. Monaco F, Vignapiano A, Piacente M, et al. Innova4Health: an integrated approach for prevention of recurrence and personalized treatment of major depressive disorder. Front Artif Intell. 2024;7:1366055. doi:10.3389/frai.2024.1366055
4. Jensen KHR, Dam VH, Ganz M, et al. Deep phenotyping towards precision psychiatry of first-episode depression: the Brain Drugs-Depression cohort. BMC Psychiatry. 2023. doi:10.1186/s12888-023-04618-x
5. Amirhosseini MH, Ayodele AL, Karami A. Prediction of depression severity and personalised risk factors using machine learning on multimodal data. In: 2024 IEEE 12th

International Conference on Intelligent Systems (IS); 2024; Varna, Bulgaria. p. 1-7. doi:10.1109/IS61756.2024.10705185
6. Shani R, Tal S, Derakshan N, et al. Personalized cognitive training: protocol for individual-level meta-analysis implementing machine learning methods. J Psychiatr Res. 2021;138:342–348. doi:10.1016/j.jpsychires.2021.03.043
7. J R, Vijayaraghavan A, K R A, et al. AI powered chatbot for mental health treatment. In: 2024 First International Conference on Technological Innovations and Advance Computing (TIACOMP); 2024; Bali, Indonesia. p. 168–172. doi:10.1109/TIACOMP64125.2024.00037
8. Pandey S, Sharma S, Wazir S. Mental healthcare chatbot based on natural language processing and deep learning approaches: Ted the therapist. Int J Inf Technol. 2022;14:3757–3766. doi:10.1007/s41870-022-00999-6
9. Ravichand M, Singh J, Shelke NA, et al. Evaluating the efficacy of deep learning models in personalizing treatment for anxiety disorders. In: 2024 4th International Conference on Intelligent Technologies (CONIT); 2024; Bangalore, India. p. 1–6. doi:10.1109/CONIT61985.2024.10626514
10. R K, Priyanka S, P S, et al. AI-driven approaches to enhancing mental wellbeing and stress relief. In: 2025 International Conference on Multi-Agent Systems for Collaborative Intelligence (ICMSCI); 2025; Erode, India. p. 925–931. doi:10.1109/ICMSCI62561.2025.10894494
11. K N, J U. MediBot: healthcare assistant on mental health and well being. In: 2023 7th International Conference on Computation System and Information Technology for Sustainable Solutions (CSITSS); 2023; Bangalore, India. p. 1–5. doi:10.1109/CSITSS60515.2023.10334083
12. de Filippis R, Foysal AA. Advanced machine learning models for gender-specific antidepressant response prediction overcoming data imbalance for precision psychiatry. Sci Res. 2025. doi:10.4236/oalib.1112895
13. Hornstein S, Forman-Hoffman V, Nazander A, et al. Predicting therapy outcome in a digital mental health intervention for depression and anxiety: a machine learning approach. Digit Health. 2021;7. doi:10.1177/20552076211060659
14. 1 Lipschitz JM, Lin S, Saghafian S, et al. Digital phenotyping in bipolar disorder: using longitudinal Fitbit data and personalized machine learning to predict mood symptomatology. Acta Psychiatr Scand. 2025;151(3):434–447. doi:10.1111/acps.13765
15. Eid MM, Yundong W, Benneh Mensah G, et al. Treating psychological depression utilising artificial intelligence: AI for precision medicine – focus on procedures. Mesopotam J Artif Intell Healthc. 2023;2023:76–81. doi:10.58496/MJAIH/2023/015

16. Shah RV, Grennan G, Zafar-Khan M, et al. Personalized machine learning of depressed mood using wearables. Transl Psychiatry. 2021;11:338. doi:10.1038/s41398-021-01445-0
17. Benrimoh D, Kleinerman A, Furukawa TA, et al. Towards outcome-driven patient subgroups: a machine learning analysis across six depression treatment studies. Am J Geriatr Psychiatry. 2024;32(3):280–292. doi:10.1016/j.jagp.2023.09.009
18. Meinlschmidt G, Tegethoff M, Belardi A, et al. Personalized prediction of smartphone-based psychotherapeutic micro-intervention success using machine learning. J Affect Disord. 2020;264:430–437. doi:10.1016/j.jad.2019.11.071
19. Doe J, Smith A, Lee B. MUBS: a personalized recommender system for behavioral activation in mental health. In: Proceedings of the 2020 CHI Conference on Human Factors in Computing Systems; 2020. p. 1–12. doi:10.1145/3313831.3376795
20. Johnson K, Williams M, Zhao Y. Personalized recommendations in mental health apps: the impact of autonomy and data sharing. In: Proceedings of the 2021 CHI Conference on Human Factors in Computing Systems; 2021. p. 1–13. doi:10.1145/3411764.3445678
21. Chen L, Kim H, Patel R. Exploring the effects of AI-assisted emotional support processes in online mental health communities. In: Extended Abstracts of the 2022 CHI Conference on Human Factors in Computing Systems; 2022. p. 1–8. doi:10.1145/3491101.3503658
22. Alslaity A, Chan G, Orji R, et al. Insights from longitudinal evaluation of Moodie mental health app. In: Extended Abstracts of the 2022 CHI Conference on Human Factors in Computing Systems (CHI EA '22); 2022. Article 308. p. 1–8. doi:10.1145/3491101.3519851
23. Sharma MK, Nachappa MN, Kumar R. Personalized treatment recommendations for mental health disorders using AI and big healthcare data. In: 2023 IEEE International Conference on ICT in Business Industry & Government (ICTBIG); 2023; Indore, India. p. 1–6. doi:10.1109/ICTBIG59752.2023.10455991
24. Webb CA, Hirshberg MJ, Davidson RJ, et al. Personalized prediction of response to smartphone-delivered meditation training: randomized controlled trial. J Med Internet Res. 2022;24(11):e41566. doi:10.2196/41566
25. van Bronswijk SC, DeRubeis RJ, Lemmens LHJM, et al. Precision medicine for long-term depression outcomes using the Personalized Advantage Index approach: cognitive therapy or interpersonal psychotherapy? *Psychol Med*. 2021;51(2):279–289. doi:10.1017/S0033291719003192
26. Ramzan HA, Abdulah F, Ahmad M, et al. AI-driven personalization of e-therapy interventions for anxiety, stress, and depression. In: *2024 18th International Conference on Open Source Systems and Technologies (ICOSST)*; 2024; Lahore, Pakistan. p. 1–6. doi:10.1109/ICOSST64562.2024.10871158

27. Schmidt F, Hammerfald K, Jahren HH, et al. Using machine learning to recommend personalized modular treatments for common mental health disorders. In: 2023 IEEE International Conference on Digital Health (ICDH); 2023; Chicago, IL, USA. p. 150–157. doi:10.1109/ICDH60066.2023.00030
28. Pushpa G, Chaitra M, Kolur LP, et al. An advanced AI framework for mental health diagnostics using bidirectional encoder representations from transformers with gated recurrent units and convolutional neural networks. *Ing Sci Int J Inf Syst Intell*. 2025;30(1):213–220. doi:10.18280/isi.300118
