# Supplementary material for: AI-Driven Mental Health Support for Caregivers of Individuals With Alzheimer Disease: Systematic Literature Review and Development of a Conceptual Framework
Source: JMIR Ment Health. 2026 Mar 6;13:e79973. doi: 10.2196/79973 (PMC13005065; doi:10.2196/79973)
Supplement: Multimedia Appendix 5 [file mental_v13i1e79973_app5.docx]

Table 3. Detailed explainability, clinical Integration, and AI decision impact

| **Study** | **AI Explanation Methods (Explainability & Interpretability Techniques)** | **AI System Design & Implementation Challenges** | **Model Performance vs. Explainability Trade-offs** | **Clinician-Centered Challenges & Real-World Implementation** | **AI Impact on Clinical Decision-Making & Patient Outcomes** |
| --- | --- | --- | --- | --- | --- |
| [[1]](#Bookmark1) | Random Forest model to avoid overfitting due to its predictive power | Data variability due to length of therapy sessions, different selection algorithms yielding different results | Models predicting symptoms such as delusions and hallucinations were not examined. | Limited use to Clinical Psychotherapists during sessions and treatment. | The model was proven clinically applicable to help identify patients with major depression |
| [[2]](#Bookmark3) | N/A | N/A | N/A | N/A | Effective identification of Patients with Major Depressive Disorder (MDD) |
| [[3]](#Bookmark3) | N/A | N/A | N/A | Adjustment to sensitivity of the algorithm | Clinically Applicable to identify patients with severe and major depression. |
| [[4]](#Bookmark4) | N/A | N/A | N/A | N/A | N/A |
| [[5]](#Bookmark5) | XGBoost, SVM, LR, RF, and Lasso for Literature record of accuracy, interpretability, complexity, and computational efficiency | N/A | N/A | N/A | Algorithms proved effective in predicting depression severity and personalized risk factors, giving potential to personalized intervention approaches |
| [[6]](#Bookmark6) | SelectKBest feature selection method to enhance algorithm interoperability and streamline dataset complexity | N/A | N/A | Efficacy of the intervention models | N/A |
| [[7]](#Bookmark7) | N/A | Response Speed, Tailored, Responses, Accuracy, Privacy and Confidentiality | N/A | Response Speed, Tailored, Responses, Accuracy, Privacy and Confidentiality | The Chatbot was effective in behaving like a virtual therapist, and continuous improvements could allow it to be more clinically applicable |
| [[8]](#Bookmark8) | DRL for personalization to the patient, RNN for, LSTM for interpreting when treatment is effective and when signs of diseases begin to appear. | Accuracy of the Deep learning models | N/A | N/A | The Deep Learning Models Showed great effectiveness and accuracy, allowing for healthcare professionals to give more accurate and personalized treatment |
| [[9]](#Bookmark9) | NLU, NLP, and NER | The scope of the chatbots’ treatment, privacy | N/A | None of the chatbots have been clinically tested in trials | The AI-based technologies have shown particularly good results and potential. |
| [[10]](#Bookmark10) | N/A | N/A | N/A | N/A | N/A |
| [[11]](#Bookmark11) | N/A | N/A | N/A | N/A | N/A |
| [[12]](#Bookmark12) | Gradient Boosting Classifier, Feature importance analysis, Confusion matrix, ROC Curve, Exploratory Data Visualization (Pair Plot) | Imbalanced datasets due to response variability to anti-depressants | Higher accuracy when class imbalance is addressed with SMOTE application to the datasets prior to model training, necessity for multidimensional data integration, inclusion of additional variables (e.g., genetic and hormonal markers) | lack of explainability due to various other factors such as genomic, neuro imaging, hormonal data, treatment adherence | Tailored antidepressant recommendations based on patient demographics and clinical metrics |
| [[13]](#Bookmark13) | Exhaustive grid search, Sklearns SelectKBest | Missing data, variability in test set and train set, lack of realistic baseline, conservative approach on hyperparameter tuning and feature selection | Missing data lowered the predictive performance of the model | The chosen approach lacks a realistic baseline because where easily interpretable data such as questionnaires are used, it might be possible that human clinicians can predict better than the model | Tailored healthcare by predicting patient’s response to treatment |
| [[14]](#Bookmark14) | Random forest portion in the Binary Mixed Model Forest algorithm | Fitbit compliance challenges | Expects high Fitbit compliancy and timely completion of self-reported PHQ-8 and ASRM measures | Wearable Device constraint (heavily dependent on Fitbit generated data) | Personalized mood symptomatology prediction |
| [[15]](#Bookmark15) | N/A | N/A | N/A | N/A | N/A |
| [[16]](#Bookmark16) | Multimodal utilization and best model selection using voting regression (low MAPE among models), SHAP (SHapley Additive Explanations) for feature importance in patient predictions | Data variability due to different device manufacturers, missing data issues, sensor variability challenges | Model was developed using low sample size (14 participants) and may have explainability challenges when expanded to real world population | Wearable device constraint (dependent on Smasung wristwatch data) and type of EEG utilization (in real world implementation, clinicians might use a different sensor other than wireless EEG), data integration of all the features collected (43 features in this case) | Personalized multimodal depression treatment |
| [[17]](#Bookmark17) | Distributions for key features, Cluster Statistical analysis, visualization | Paper states that this model is not yet ready for clinical implementation | Limited number of features, lack of key socio-demographics features, lack of data from different treatment options | Focuses only on pharmacological treatment options, (missing explanation for other treatment modalities) | Better clinical interpretability |
| [[18]](#Bookmark18) | R for data analyses, Visualizations, statistical testing, lme4, optimx, car, dplyr, Imertest, tidyr, haven, ggplot2, caret, bdpv, PMCMR, vcrpart | Model overfitting issues when increasing number of predictors | The RF approach does not allow firm conclusions about the exact contribution of each factor on model’s predictability. Lack of explainability on predictable differences between psychotherapeutic techniques | Studies were conducted on subjects who have no history of cardiovascular or neurological or mental disorders. This raises questions of its implementation in real world clinicians use. Study was ethnicity specific (Korean) | Personalized prediction of mood changes in male adults |
| [[19]](#Bookmark19) | Personalized Recommendations via Naive Bayes | The recommender model did not include any post-filtering functionality on the recommended activities. Difficulty in maintaining engagement, lack of long-term adherence in users | Trade-off between Personalized Recommendations and Transparency   Higher personalization improved engagement but required user feedback (ratings) to refine recommendations | Not integrated into clinician workflows; designed for self-management by patients   MUBS does not incorporate the support of a therapist | Positive impact on daily activity planning, improved self-awareness of behavior, reduction in depressive symptom |
| [[20]](#Bookmark20) | Naïve Bayes classifier for activity suggestions. | Users were reluctant to share mobile sensor data, making it difficult to train AI models for true personalization. | Higher AI complexity could have improved personalization, but at the cost of making AI decision-making less interpretable to users   Users perceived AI recommendations as personalized, even though they were randomized in a placebo-controlled study | Health professionals questioned the reliability of AI-generated recommendations that lacked a clear clinical basis.   Not tested in collaboration with human therapists making it difficult to assess its true effectiveness in clinical settings. | Limited direct clinical impact = AI was not designed for medical diagnosis, but helped improve user engagement in digital mental health interventions. |
| [[21]](#Bookmark21) | ELECTRA (Efficiently Learning an Encoder that Classifies Token Replacements Accurately), KRWordRank for keyword extraction | sometimes misclassified distress signals, leading to inappropriate or ineffective emotional support.   The AI model was trained primarily on structured emotional data, which did not always reflect the diversity of real-world emotional expression. | AI was accurate in classifying straightforward emotional expressions but struggled with subtle emotional nuances.   Users could not see how AI determined their emotional state = Less explainability.  Using deep learning models (ELECTRA, GPT-2, LSTM) improved emotion classification accuracy, but at the cost of reduced interpretability | Clinicians raised concerns about AI misclassifying user distress, potentially leading to harmful or inappropriate interventions.   AI's lack of accountability  AI was used solely in peer-support settings and was not integrated with licensed therapists, limiting its clinical applicability. | Supplementary to human support, not a replacement of human intervention. |
| [[22]](#Bookmark22) | Random Forest and XGBoost models | Over time, users changed their mood-logging habits, making long-term predictions more complex. Some users felt "over-predicted" by AI, leading to distrust in system recommendations. | AI-driven mood predictions were highly accurate in the short term, but long-term trends required adjustments,   Users did not understand how AI adapted recommendations based on seasonal patterns.   The Random Forest model provided high accuracy but was not interpretable. | Clinicians recognized AI’s potential for early mood disorder detection but required better explainability in mood predictions.   Mental health professionals found it difficult to validate AI-driven mood assessments, as explanations were unclear.   Clinicians questioned whether AI should be relied upon for mood disorder intervention without human oversight. | N/A |
| [[23]](#Bookmark23) | Deep Learning for Diagnosis (DL-D), Collaborative Filtering for Treatment Recommendation (CF –TR), and Reinforcement Learning for Medication (RL- M) | N/A | N/A | N/A | Algorithms proved effective in accurate diagnosis, and doctors can use these models to ensure better treatment outcomes. |
| [[24]](#Bookmark24) | Random Forest-Based imputation for missing data and two regressive models for outcome prediction | N/A | N/A | Other Variables and Characteristics not considered and/or excluded | The models proved to perform better than predicted, giving them significant potential. |
| [[25]](#Bookmark25) | Random forest | None mentioned | None mentioned | None mentioned | Depression severity |
| [[26]](#Bookmark26) | N/A | N/A | N/A | N/A | N/A |
| [[27]](#Bookmark27) | Braive’s system | none | None mentioned | None mentioned | Provide treatment options based on pre therapy assessment |
| [[28]](#Bookmark28) | BERT, GRU and CNN | Not specified | Not specified | Not specified | Not specified |

**References:**

1. Kim K, Ryu JI, Lee BJ, et al. A machine-learning-algorithm-based prediction model for psychotic symptoms in patients with depressive disorder. J Pers Med. 2022;12(8):1218. doi:10.3390/jpm12081218
2. Rubel JA, Zilcha-Mano S, Giesemann J, et al. Predicting personalized process-outcome associations in psychotherapy using machine learning approaches: A demonstration. Psychother Res. 2020;30(3):300–309. doi:10.1080/10503307.2019.1597994
3. Monaco F, Vignapiano A, Piacente M, et al. Innova4Health: an integrated approach for prevention of recurrence and personalized treatment of major depressive disorder. Front Artif Intell. 2024;7:1366055. doi:10.3389/frai.2024.1366055
4. Jensen KHR, Dam VH, Ganz M, et al. Deep phenotyping towards precision psychiatry of first-episode depression: the Brain Drugs-Depression cohort. BMC Psychiatry. 2023. doi:10.1186/s12888-023-04618-x
5. Amirhosseini MH, Ayodele AL, Karami A. Prediction of depression severity and personalised risk factors using machine learning on multimodal data. In: 2024 IEEE 12th International Conference on Intelligent Systems (IS); 2024; Varna, Bulgaria. p. 1-7. doi:10.1109/IS61756.2024.10705185
6. Shani R, Tal S, Derakshan N, et al. Personalized cognitive training: protocol for individual-level meta-analysis implementing machine learning methods. J Psychiatr Res. 2021;138:342–348. doi:10.1016/j.jpsychires.2021.03.043
7. J R, Vijayaraghavan A, K R A, et al. AI powered chatbot for mental health treatment. In: 2024 First International Conference on Technological Innovations and Advance Computing (TIACOMP); 2024; Bali, Indonesia. p. 168–172. doi:10.1109/TIACOMP64125.2024.00037
8. Pandey S, Sharma S, Wazir S. Mental healthcare chatbot based on natural language processing and deep learning approaches: Ted the therapist. Int J Inf Technol. 2022;14:3757–3766. doi:10.1007/s41870-022-00999-6
9. Ravichand M, Singh J, Shelke NA, et al. Evaluating the efficacy of deep learning models in personalizing treatment for anxiety disorders. In: 2024 4th International Conference on Intelligent Technologies (CONIT); 2024; Bangalore, India. p. 1–6. doi:10.1109/CONIT61985.2024.10626514
10. R K, Priyanka S, P S, et al. AI-driven approaches to enhancing mental wellbeing and stress relief. In: 2025 International Conference on Multi-Agent Systems for Collaborative Intelligence (ICMSCI); 2025; Erode, India. p. 925–931. doi:10.1109/ICMSCI62561.2025.10894494
11. K N, J U. MediBot: healthcare assistant on mental health and well being. In: 2023 7th International Conference on Computation System and Information Technology for Sustainable Solutions (CSITSS); 2023; Bangalore, India. p. 1–5. doi:10.1109/CSITSS60515.2023.10334083
12. de Filippis R, Foysal AA. Advanced machine learning models for gender-specific antidepressant response prediction overcoming data imbalance for precision psychiatry. Sci Res. 2025. doi:10.4236/oalib.1112895
13. Hornstein S, Forman-Hoffman V, Nazander A, et al. Predicting therapy outcome in a digital mental health intervention for depression and anxiety: a machine learning approach. Digit Health. 2021;7. doi:10.1177/20552076211060659
14. 1 Lipschitz JM, Lin S, Saghafian S, et al. Digital phenotyping in bipolar disorder: using longitudinal Fitbit data and personalized machine learning to predict mood symptomatology. Acta Psychiatr Scand. 2025;151(3):434–447. doi:10.1111/acps.13765
15. Eid MM, Yundong W, Benneh Mensah G, et al. Treating psychological depression utilising artificial intelligence: AI for precision medicine – focus on procedures. Mesopotam J Artif Intell Healthc. 2023;2023:76–81. doi:10.58496/MJAIH/2023/015
16. Shah RV, Grennan G, Zafar-Khan M, et al. Personalized machine learning of depressed mood using wearables. Transl Psychiatry. 2021;11:338. doi:10.1038/s41398-021-01445-0
17. Benrimoh D, Kleinerman A, Furukawa TA, et al. Towards outcome-driven patient subgroups: a machine learning analysis across six depression treatment studies. Am J Geriatr Psychiatry. 2024;32(3):280–292. doi:10.1016/j.jagp.2023.09.009
18. Meinlschmidt G, Tegethoff M, Belardi A, et al. Personalized prediction of smartphone-based psychotherapeutic micro-intervention success using machine learning. J Affect Disord. 2020;264:430–437. doi:10.1016/j.jad.2019.11.071
19. Doe J, Smith A, Lee B. MUBS: a personalized recommender system for behavioral activation in mental health. In: Proceedings of the 2020 CHI Conference on Human Factors in Computing Systems; 2020. p. 1–12. doi:10.1145/3313831.3376795
20. Johnson K, Williams M, Zhao Y. Personalized recommendations in mental health apps: the impact of autonomy and data sharing. In: Proceedings of the 2021 CHI Conference on Human Factors in Computing Systems; 2021. p. 1–13. doi:10.1145/3411764.3445678
21. Chen L, Kim H, Patel R. Exploring the effects of AI-assisted emotional support processes in online mental health communities. In: Extended Abstracts of the 2022 CHI Conference on Human Factors in Computing Systems; 2022. p. 1–8. doi:10.1145/3491101.3503658
22. Alslaity A, Chan G, Orji R, et al. Insights from longitudinal evaluation of Moodie mental health app. In: Extended Abstracts of the 2022 CHI Conference on Human Factors in Computing Systems (CHI EA '22); 2022. Article 308. p. 1–8. doi:10.1145/3491101.3519851
23. Sharma MK, Nachappa MN, Kumar R. Personalized treatment recommendations for mental health disorders using AI and big healthcare data. In: 2023 IEEE International Conference on ICT in Business Industry & Government (ICTBIG); 2023; Indore, India. p. 1–6. doi:10.1109/ICTBIG59752.2023.10455991
24. Webb CA, Hirshberg MJ, Davidson RJ, et al. Personalized prediction of response to smartphone-delivered meditation training: randomized controlled trial. J Med Internet Res. 2022;24(11):e41566. doi:10.2196/41566
25. van Bronswijk SC, DeRubeis RJ, Lemmens LHJM, et al. Precision medicine for long-term depression outcomes using the Personalized Advantage Index approach: cognitive therapy or interpersonal psychotherapy? *Psychol Med*. 2021;51(2):279–289. doi:10.1017/S0033291719003192
26. Ramzan HA, Abdulah F, Ahmad M, et al. AI-driven personalization of e-therapy interventions for anxiety, stress, and depression. In: *2024 18th International Conference on Open Source Systems and Technologies (ICOSST)*; 2024; Lahore, Pakistan. p. 1–6. doi:10.1109/ICOSST64562.2024.10871158
27. Schmidt F, Hammerfald K, Jahren HH, et al. Using machine learning to recommend personalized modular treatments for common mental health disorders. In: 2023 IEEE International Conference on Digital Health (ICDH); 2023; Chicago, IL, USA. p. 150–157. doi:10.1109/ICDH60066.2023.00030
28. Pushpa G, Chaitra M, Kolur LP, et al. An advanced AI framework for mental health diagnostics using bidirectional encoder representations from transformers with gated recurrent units and convolutional neural networks. *Ing Sci Int J Inf Syst Intell*. 2025;30(1):213–220. doi:10.18280/isi.300118
